# The Remaining Conundrum of the Role of the Na^+^/H^+^ Exchanger Isoform 1 (NHE1) in Cardiac Physiology and Pathology: Can It Be Rectified?

**DOI:** 10.31083/j.rcm2308284

**Published:** 2022-08-15

**Authors:** Morris Karmazyn, Grant N. Pierce, Larry Fliegel

**Affiliations:** ^1^Department of Physiology and Pharmacology, University of Western Ontario, London, ON N6A 5C1, Canada; ^2^Institute of Cardiovascular Sciences, Albrechtsen Research Centre, St. Boniface Hospital, and Department of Physiology and Pathophysiology, Rady Faculty of Health Sciences, University of Manitoba, Winnipeg, MB R2H 2A6, Canada; ^3^Department of Biochemistry, University Alberta, Edmonton, AB T6G 2H7, Canada

**Keywords:** NHE1 regulation, NHE1 inhibitors, cardiac hypertrophy and remodelling, ischemia/reperfusion injury, pyrazinoyl guanidine

## Abstract

The mammalian ${\mathrm{Na}}^{+}$${\mathrm{/H}}^{+}$ exchanger (NHE) is a family of ubiquitous membrane proteins present in humans. Isoform one (NHE1) is present on the plasma membrane and regulates intracellular pH by removal of one intracellular proton in exchange for one extracellular sodium thus functioning as an electroneutral process. Human NHE1 has a 500 amino acid membrane domain plus a C-terminal 315 amino acid, regulatory cytosolic tail. It is regulated through a cytosolic regulatory C-terminal tail which is subject to phosphorylation and is modulated by proteins and lipids. Substantial evidence has implicated NHE1 activity in both myocardial ischemia and reperfusion damage and myocardial remodeling resulting in heart failure. Experimental data show excellent cardioprotection with NHE1 inhibitors although results from clinical results have been mixed. In cardiac surgery patients receiving the NHE1 inhibitor cariporide, subgroups showed beneficial effects of treatment. However, in one trial this was associated with a significantly increased incidence of ischemic strokes. This likely reflected both inappropriate dosing regimens as well as overly high drug doses. We suggest that further progress towards NHE1 inhibition as a treatment for cardiovascular disease is warranted through the development of novel compounds to inhibit NHE1 that are structurally different than those previously used in compromised clinical trials. Some novel pyrazinoyl guanidine inhibitors of NHE1 are already in development and the recent elucidation of the three-dimensional structure of the NHE1 protein and identity of the inhibitor binding site may facilitate development. An alternative approach may also be to control the endogenous regulation of activity of NHE1, which is activated in disease.

## 1. Introduction

**Fig. 1.**
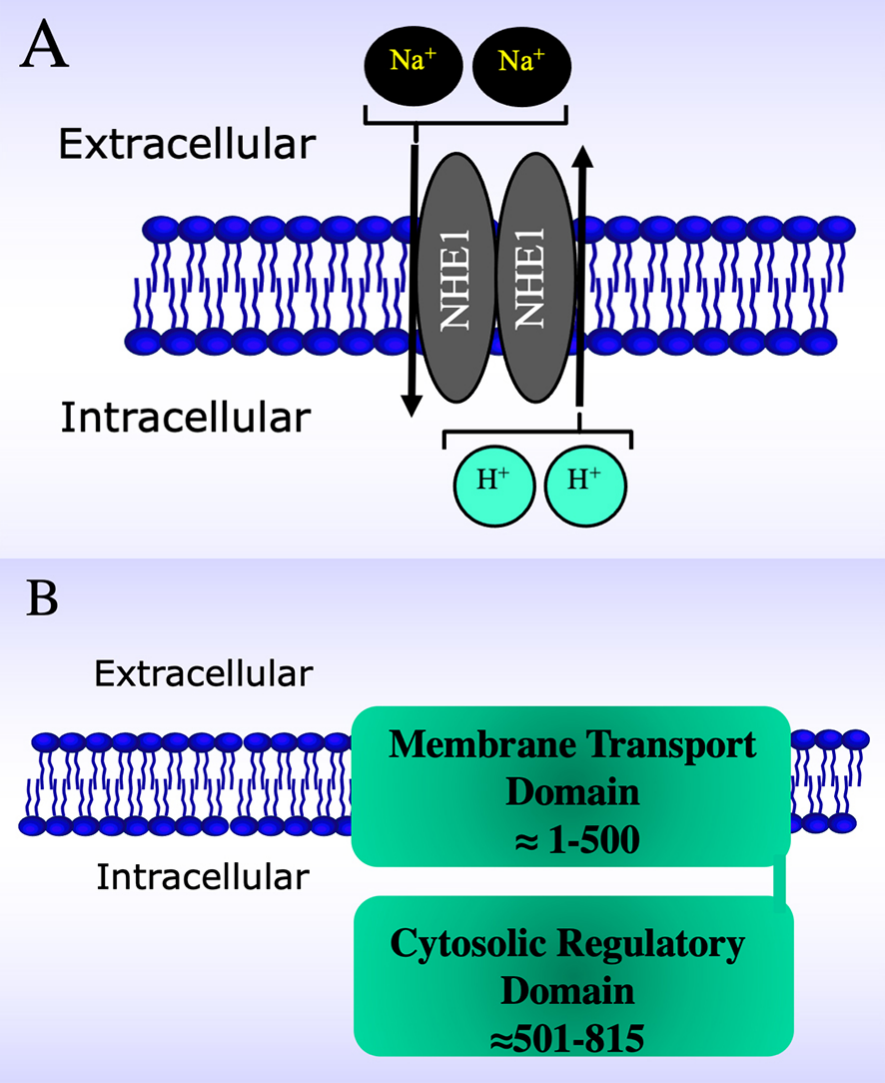
**Schematic diagrams of the ${\mathrm{Na}}^{+}$${\mathrm{/H}}^{+}$ exchanger (NHE1) within the plasma membrane**. (A) Schematic diagram illustrating dimeric structure of NHE1 within a lipid bilayer. Arrows indicate direction of transport. (B) Schematic diagram of NHE1 withing the lipid bilayer illustrating the two-domain structure, and approximate locations within the membrane.

The ubiquitously expressed mammalian ${\mathrm{Na}}^{+}$${\mathrm{/H}}^{+}$ exchanger (NHE) is a family of membrane proteins of human cells of which there are currently 10 known isoforms. Isoform one (NHE1) removes a single intracellular proton in exchange for a single extracellular sodium ion (Fig. 1A) and is ubiquitously present throughout the tissues and cell types of the body [1, 2]. NHE maintains intracellular pH (${\mathrm{pH}}_{\text{𝑖}}$), thus protecting cells from acidification which results from metabolism. It also responds to osmotic challenge regulating cell volume [3, 4]. There are nine SLC9A type isoforms of NHE, two SLC9B types and also two SLC9C types. Most isoforms of NHEs have restricted cellular locations or intracellular locations but NHE1 (SLC9A1) is the primary plasma membrane isoform found in virtually all mammalian cells [4, 5, 6, 7, 8, 9, 10]. NHE1 consists of two general domains. One is the membrane transport domain which moves ions, and the second is a regulatory cytosolic domain (Fig. 1B). The human N-terminal membrane transport domain is approximately 500 amino acids and its atomic structure has recently been determined [11]. The human cytosolic regulatory domain is an additional 315 amino acids that functions to regulate the membrane domain [12].

The physiological and pathological roles of NHE1 are many. Outside of the myocardium NHE1 plays a role in cell growth, proliferation and differentiation [2, 13, 14, 15, 16]. NHE1 is also an important trigger of growth and metastasis in cancer, notably as a trigger of metastasis in breast cancer [17, 18, 19, 20, 21]. Genetic mutations in NHE1 and its absence, have been shown to be responsible for the disease Lichtenstein-Knorr syndrome which manifests itself through many developmental defects and ataxia and hearing loss [22, 23].

**Fig. 2.**
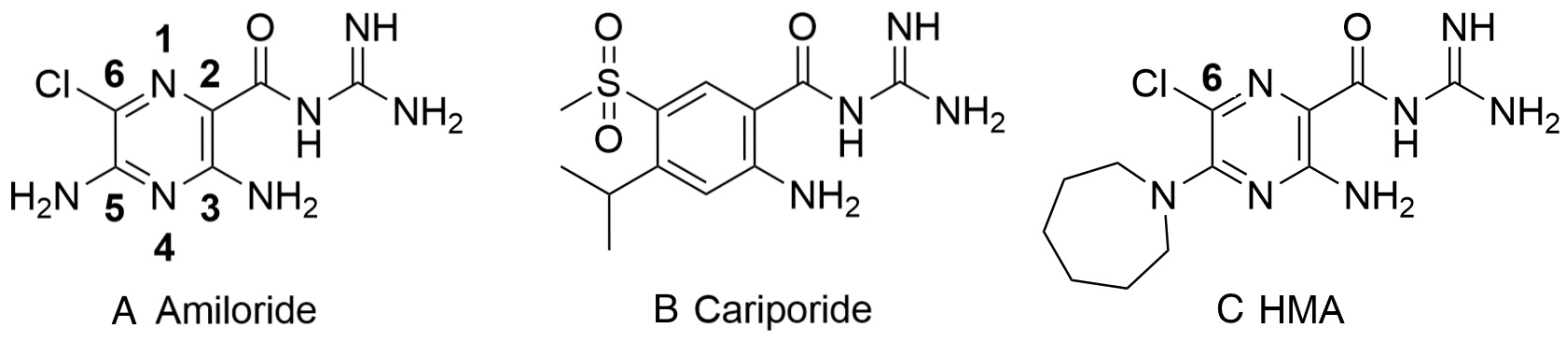
**Structure of amiloride, cariporide and hexamethylene amiloride (HMA) (A-C, respectively)**. Amiloride and HMA are pyrazinoyl guanidines that differ in the aromatic core from cariporide a benzoyl guanidine.

In the myocardium, NHE1 is the only plasma membrane isoform of ${\mathrm{Na}}^{+}$${\mathrm{/H}}^{+}$ exchanger present. Its activity was demonstrated as early as 1984–1985 [24, 25, 26, 27, 28] and a human clone was initially isolated from the myocardium in 1993 [29]. NHE1 is associated with both ischemic reperfusion damage to the myocardium and heart hypertrophy and its inhibition shows beneficial effects in animal models of this disease [30, 31, 32, 33]. It is inhibited by pyrazinoyl guanidines including amiloride, a potassium-sparing diuretic used for decades to treat hypertension and heart failure (in combination with other drugs). NHE1 is also inhibited by benzoyl guanidines such as cariporide which were later developed for clinical experimentation (Fig. 2A). Despite its key role in cardiac physiology and pathology, there is as yet no known NHE1-based therapy developed for clinical protection of the myocardium. Why is that?

This review presents a discussion of the structure, chemistry and regulation of NHE1 in the myocardium as well as location of inhibitor binding sites and the potential development of novel NHE1 inhibitors. This is followed by assessment of the role of NHE1 in cardiac pathologies including ischemic and reperfusion injury, myocardial hypertrophy and remodeling resulting in heart failure as well as its role in diabetes related cardiac pathology. Finally, we discuss the clinical potential of NHE1 inhibitors to treat heart disease reflecting on completed, ongoing and future clinical trials. Our goal is to both update the field and to stimulate potential development of new inhibitors that can be useful in treating heart disease and indeed other common human afflictions.

## 2. Expression and Localization of Myocardial NHE1

NHE1 plays an important physiological role in regulating intracellular pH (${\mathrm{pH}}_{\text{𝑖}}$) in the myocardium. Through the generation of protons by intermediary metabolism, and also because of the negative membrane potential, protons accumulate within the cytosol and inhibit contractility. NHE1 removes these protons. Cardiac NHE1 has distinct activity characteristics, having a very steep relationship between ${\mathrm{pH}}_{\text{𝑖}}$ and activity [34]. While ${\mathrm{HCO}}_{3}$^-^ based transporters can also contribute partially to recovery from intracellular proton accumulation [35, 36, 37, 38, 39, 40, 41], as can lactate proton symport [42], so when NHE1 is inhibited or absent, these other mechanisms can aid in pH recovery from acidosis. Additionally, evaluation of the relative contribution of bicarbonate dependent and the NHE transporter to acid extrusion showed that NHE1 is the dominant transporter for proton efflux following intracellular acid load [43]. NHE1 appears to be the principal pH regulatory mechanism in cardiomyocytes. It is the only plasma membrane isoform present in the myocardium that localizes to the intercalated disks and transverse tubules [44, 45]. Cardiac cells do not possess NHE2–5 [46, 47, 48, 49] and NHE6–9 are localized to intracellular organelle membranes such as mitochondria, endosomes and the Golgi network so they do not directly contribute to proton extrusion from cardiomyocytes [50, 51]. cDNA for NHE1 codes for the identical NHE1 message as in other tissues [29] and though a different size mRNA for NHE1 has been shown to occur in ischemic conditions [52], this does not code for a functional protein. ${\mathrm{Na}}^{+}$${\mathrm{/H}}^{+}$ exchange has been clearly demonstrated in cardiac sarcolemma vesicles where it was inhibited by amiloride [28, 53]. The level of NHE1 protein is low, similar to other tissues, but it is clearly very active. It has been possible to immunoprecipitate NHE1 *in vivo* from isolated cardiomyocytes and tissue, and *in vivo* phosphorylation was demonstrated [54, 55]. Expression levels of NHE1 in the heart can vary. Ischemia, with or without reperfusion, increases NHE1 mRNA up to seven-fold [56, 57]. Expression in the myocardium also varies developmentally. In rabbit fetal and neonatal hearts mRNA levels are elevated [58]. These results correlate well with gene expression from the NHE1 promoter which was examined in transgenic mice and showed that NHE1 transcription was maximum in the heart and liver in 12-day-old embryonic mice [59].

## 3. Regulation of NHE1 in the Myocardium

### 3.1 Hormonal Regulation

**Fig. 3.**
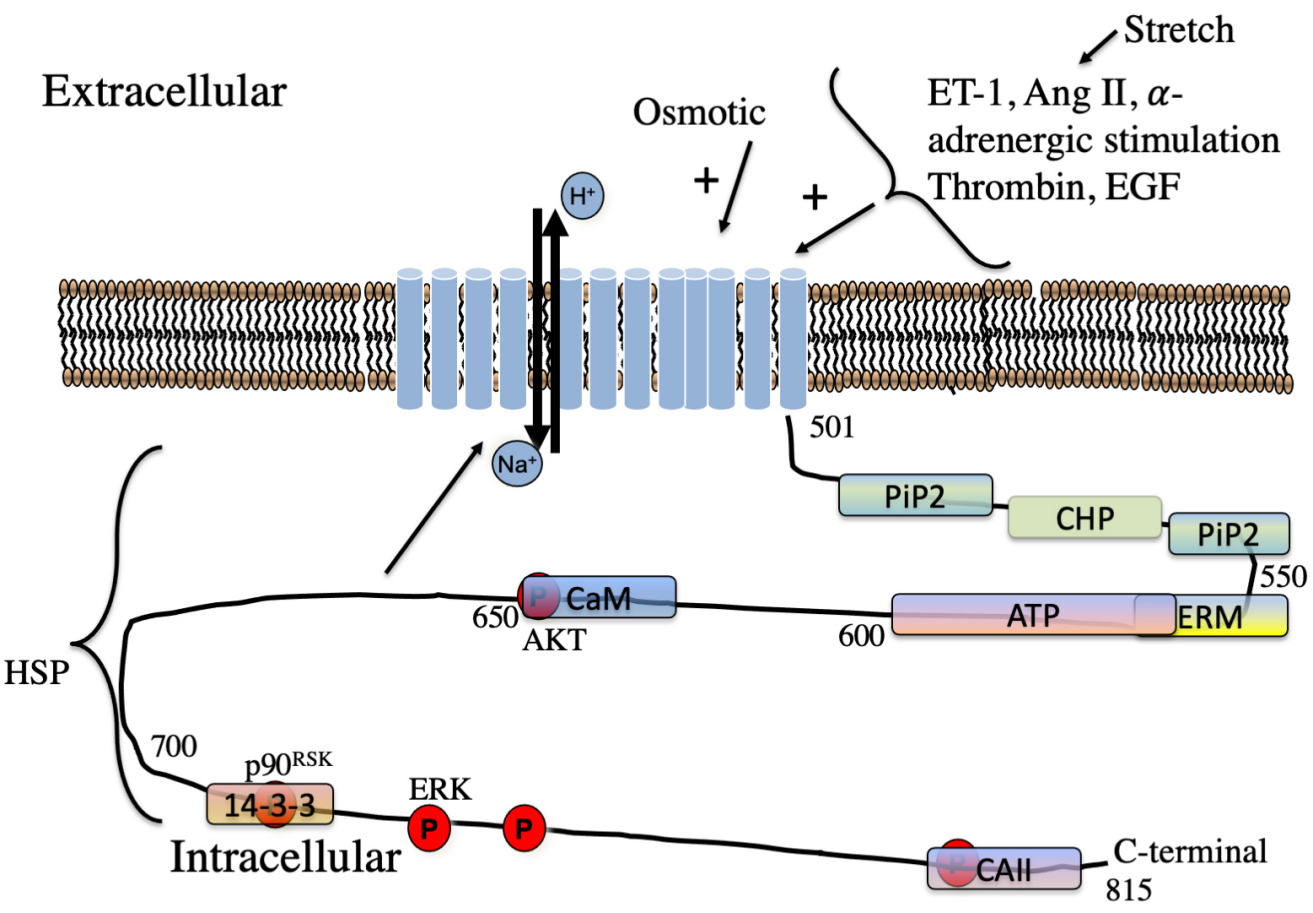
**Schematic illustration of regulators of NHE1 in the myocardium**. Hormones regulating NHE1 are indicated. The approximate location of lipid, protein and phosphorylation sites on the cytosolic regulatory tail is indicated. P, phosphorylation sites. The kinases phosphorylation sites of AKT, ERK and ${\mathrm{p90}}^{\text{RSK}}$ sites are shown. CaM, Calmodulin; CHP, calcineurin homologous protein; 14-3-3,14-3-3 protein; HSP, heat shock protein. Some sites overlap.

NHE1 is normally quiescent in the myocardium at neutral pH, however the protein is activated when intracellular pH decreases and is also activated by stimuli such as growth factors, hormones and osmotic stress. These tend to shift the activity curve such that the protein is active at more alkaline ${\mathrm{pH}}_{\text{𝑖}}$. Regulation of NHE1 occurs in all tissues. This review is restricted in large part to regulation of NHE1 in the myocardium (see also reviews in [12, 60]. Regulation of NHE1 in the myocardium is extremely important. Evidence has shown that activating NHE1 activity through changes in regulation of the protein, accentuate NHE1-induced damage to the myocardium [61, 62, 63, 64, 65]. As noted above, NHE1 activity and mRNA levels are elevated by myocardial ischemia, with or without reperfusion [56, 57] and this may exacerbate NHE1’s detrimental effects in disease. Additionally, targeting regulation of NHE1 has been suggested to be an important approach to treat myocardial disease [66]. Hormones and growth factors modulate cardiac NHE1activity and contribute to its role in cardiac pathology. Endothelin-I, angiotensin II, α-adrenergic agonists, thrombin, and epidermal growth factor are known to stimulate NHE1 in the myocardium. Hormonal regulation often works through activation of protein kinases that phosphorylate the regulatory cytosolic domain of NHE1. Angiotensin II and endothelin stimulate NHE1 activity and their release can occur locally after stretch [67, 68]. The stimulatory action of angiotensin II occurs *via* the ${\mathrm{AT}}_{1}$ receptor and occurs through protein kinase C and an epidermal growth factor mediated mechanism. The ${\mathrm{AT}}_{2}$ receptor mediates an opposing, counteracting inhibition of NHE1 [69]. Endothelin-1 stimulates NHE1 activity [70] as noted above, and inotropic effects of endothelin on the heart may be at least partially attributed to its stimulation of NHE1 [71]. α_1_-adrenergic agonists like phenylephrine stimulate NHE1 activity by the α_1A_-adrenoceptor/extracellular signal-regulated kinase (ERK) pathway [55, 72, 73] and this is blocked by Ras-mitogen-activated protein kinase (RSK) [74] and mitogen activated protein kinase (MAPK) [72] inhibition. NHE1 stimulation by α_1_-adrenergic agonists may also play a role in exacerbation of reperfusion-induced arrhythmias [75]. Thrombin also activates NHE1 in cardiomyocytes through a protein kinase C-mediated mechanism [76] though protein kinase C does not directly phosphorylate the NHE1 cytosolic domain [77]. Fig. 3 illustrates some of the hormones acting to stimulate NHE1.

#### 3.1.1 Hormonal Regulation of NHE1 through Kinase-Dependent Phosphorylation

Hormonal activation of NHE1 occurs at least partially through protein kinase-mediated phosphorylation or through interaction with other regulatory proteins (or lipids). While the exact percentage of activation in the myocardium that occurs through phosphorylation is not known, it has generally been estimated to be 50% of hormonal regulation though this surely varies with cell type (see reviews [12, 44]). A number of different protein kinases phosphorylate the regulatory tail which is thought to occur mainly in the C-terminal 180 amino acids [12, 60]. In brief, phosphorylation-mediated regulation of NHE1 in several tissues was described earlier [4, 10, 12, 44, 78]. Amino acids phosphorylated include Ser648 by Protein kinase B (PKB or Akt) and [79, 80] amino acids Thr718, Ser723, Ser726, Ser729 by p38 MAPK (equivalent human numbering) [81], and see also [4] for review).

The MAPK phosphorylation pathway was identified as being important in NHE1 regulation in the myocardium. This pathway is regulated by many hormones including endothelin, angiotensin II, catecholamines and some cytokines [82]. This pathway has also been shown to play a role in ischemia reperfusion activation of NHE1 leading to cardiac injury [83] and this ${\mathrm{p90}}^{\text{RSK}}$ containing pathway is activated by several other stimuli. One way to activate NHE1 in the heart is by sustained intracellular acidosis which leads to activation of Ras signaling and the kinases ERK1/2 and ${\mathrm{p90}}^{\text{RSK}}$ that directly phosphorylate the NHE1 C-terminus [55, 84, 85, 86]. The ERK1/2 and ${\mathrm{p90}}^{\text{RSK}}$ pathway has also been shown to be activated and to phosphorylate NHE1 during cardiac ischemia reperfusion injury [87]. Several studies have tried to localize the precise sites phosphorylated by the activated kinases. Early *in vitro *studies [44, 88] identified four general regions of phosphorylation of NHE1 in the cytosolic tail, (1) S693; (2) T718,S723/726/729; (3) S766/770/771; and (4) T779,S785 and of these, Ser770 and Ser771 of region three were found to mediate ERK1/2 activation of NHE1 by sustained intracellular acidosis in heart cells [55]. Additionally, Ser703 was also earlier identified as being phosphorylated by the kinase ${\mathrm{p90}}^{\text{RSK}}$ in several studies [89, 90] including a study on vascular smooth muscle cells, and this amino acid has also been suggested to be important in ischemic and reperfusion injury in the myocardium [90], though sustained intracellular acidosis can activated NHE1 independent of Ser703 and ${\mathrm{p90}}^{\text{RSK}}$ [91] (Fig. 3). A way to activate NHE1 through ERK1/2 during cardiac ischemia reperfusion, is by the elevated bursts of reactive oxygen species. Hydrogen peroxide has been shown to activate ERK1/2 and this increases phosphorylation and activation of the ${\mathrm{Na}}^{+}$${\mathrm{/H}}^{+}$ exchanger [92, 93, 94]. How phosphorylation activates NHE1 is still somewhat of a mystery, but it is clear that it results in structural changes in the cytosolic regulatory domain which somehow affect activity of the membrane domain [1, 95, 96].

Some other protein kinases also phosphorylate NHE1 though these are less well studied. Heart β-Raf protein can associate with NHE1 C-terminal domain and can phosphorylate the cytosolic tail at Thr653 [97]. Another regulatory kinase of cardiac NHE1 is PKB [79]. It phosphorylates amino acid Ser648 and this phosphorylation produces an inhibitory effect. Ser648, is within the calmodulin (CaM) high-affinity binding region (see below).

#### 3.1.2 Role of Phosphatases 

The requisite dephosphorylation of NHE1 protein must occur sometime after activation of the protein. It is not as well studied in the myocardium. Both protein phosphatases 1 and protein phosphatase 2A (PP1 and PP2A) directly associate with NHE1 [98, 99]. Colocalization of NHE1 and PP2A was shown in ventricular cardiomyocytes [98]. The calcineurin A subunit also binds to NHE1 [100], but its role in dephosphorylation of NHE1 is not yet known. Its binding may facilitate NHE1-induced translocation of NFAT and myocardial hypertrophy progression [100]. An interaction between NHE1 and the Src homology 2 domain-containing protein tyrosine phosphatase (SHP-2) has also been confirmed. Functionally, SHP-2 overexpression caused a higher steady state ${\mathrm{pH}}_{\text{𝑖}}$, and increased recovery from an acid load [101].

### 3.2 Other Regulatory Processes

#### 3.2.1 Osmotic Regulation

It is known that ${\mathrm{Na}}^{+}$${\mathrm{/H}}^{+}$ exchanger is activated by osmotic regulation. Upon exposure to hyperosmotic solutions NHE1 rapidly increases activity which results in cellular alkalinization. This is part of the regulatory volume increase in cells whereby they compensate for shrinkage that is induced by hyperosmolar external media [102]. Cardiomyocytes exposed to hyperosmolar solutions also show this osmotic activation of the ${\mathrm{Na}}^{+}$${\mathrm{/H}}^{+}$ exchanger. The effect is blocked by calmodulin antagonists and the myosin light chain kinase inhibitor ML-7 [103]. Experiments in intact hearts also show hyperosmotic activation of NHE1 which produces intracellular alkalinization [104]. During myocardial ischemia, accumulating metabolites can cause a hyperosmotic extracellular milieu [105] which could be a mechanism of activation of NHE1 *in vivo*.

#### 3.2.2 Regulation by Nitric Oxide

Nitric oxide (NO) has been shown to regulate NHE1 activity in adult ventricular cardiomyocytes in a study where which NO levels were manipulated through various approaches [106]. The response was biphasic such that NHE1 flux was activated by low NO levels but inhibited by high NO amounts. These responses were dependent on two pathways, namely a cGMP-dependent NHE1 activation and a cAMP-dependent inhibition. The protein kinases PKG and PKA were tested for their ability to phosphorylate the NHE1 C-terminus and multiple residues were phosphorylated including Ser648 and Ser703. PKA was more selective for Ser648, possibly accounting for the inhibitory effect of cAMP. The biphasic effect of nitic oxide was specific to adult cardiomyocytes and was not observed in neonatal myocytes or in MDA-MB-468 breast cancer cells [106].

#### 3.2.3 Protein-Mediated Regulation 

Protein mediated regulation of NHE1 also occurs through the cytosolic regulatory tail. Binding occurs by regulatory proteins, and also by other proteins that may be using NHE1 as a scaffold for other cellular functions aside from regulation of NHE1 activity. This regulatory mechanism has been suggested to account for 50% of the regulation of NHE1 (see reviews [12, 44]) though this is clearly difficult to quantitate and will surely vary with cell type. Most of this type of regulation has been studied in non-myocardial tissue and is briefly reviewed. The “scaffolding” of proteins may vary in response to cellular stimuli [107]. As noted above, protein phosphatases bind to the NHE1 tail and facilitate de-phosphorylation of the tail and have other physiological consequences [100]. In “opposition” to the phosphatases are kinases which also bind to the NHE1 tail. NHE1 acts as a scaffold for ERK and Raf [108]. ERK binds to the cytosolic domain at specific D-domain and F-sites binding sites. These binding sites, and the interaction of NHE1 with ERK affect not only the phosphorylation and activation of NHE1 but also the regulation and activation of ERK itself [96]. Direct binding of ERK to NHE in the myocardium has not, to our knowledge, been demonstrated.

Other interaction partners of NHE1 were reviewed [4] for all general tissues and will be briefly summarized. Several studies have also examined the “interactome” of the regulatory NHE1 cytosolic tail, describing protein that bind to the NHE1 C-terminus from the kidney [109] and from breast cancer cells [110]. Results from some of these are outlined below.

#### 3.2.4 Regulation by Calmodulin 

The calcium-binding second messenger protein known as calmodulin mediates ${\mathrm{Ca}}^{2+}$-induced activation of NHE1. It binds in the presence of ${\mathrm{Ca}}^{2+}$on two locations on the tail of NHE1. One is the high affinity binding region (amino acids 637-656) and a second is a lower, intermediate affinity region with binding at amino acids 657-700 [4, 12, 44]). Calmodulin regulates NHE1 activity through its high affinity binding site on the NHE1 tail. It binds there preventing this autoinhibitory domain from inhibiting the membrane domain. As noted above, protein kinase B phosphorylates NHE1 within the calmodulin high-affinity binding region at amino acid Ser648 (Fig. 3). This results in a reduction in NHE1 activity by preventing calmodulin binding to NHE1, and thereby preventing blocking of the autoinhibitory site on the cytosolic NHE1 tail. Snabaitis *et al*. [79] suggest that during ischemic and reperfusion injury this may be a cardioprotective mechanism. There are not many studies on the regulation of NHE1 by calmodulin in the myocardium. It has been shown that the calmodulin blocker W7, inhibits NHE1 activity in isolated cardiomyocytes [103, 111].

#### 3.2.5 Regulation by Calcineurin B Homologous Proteins 

There are several isoforms of Calcineurin B homologous proteins (CHPs, CHP1, CHP2 and CHP3) [112, 113, 114, 115]. These are ${\mathrm{Ca}}^{2+}$-binding proteins with EF-hand motifs that bind ${\mathrm{Ca}}^{2+}$ ions similar to calmodulin. CHP1 is expressed in the heart and many other tissues. CHP2 expression is mostly restricted to intestinal epithelial cells and malignant tumor cells. CHP3, was initially detected in mouse testis. It is also expressed in the heart, stomach and brain, and some specialized cells such as hematopoietic cells. CHP1 binds to the NHE1 tail at amino acids 518-537 and this binding enhances NHE1 activity [116, 117] (Fig. 3). Mutation of the CHP1 binding site causes NHE1 to have a shorter cellular half-life and causes reduced cell surface expression [118]. CHP3 also has ${\mathrm{Ca}}^{2+}$-dependent binding to NHE1. CHP3 can also enhance NHE1 stability and activity at the plasma membrane [119]. However, while CHP1 and CHP3 are both expressed in the heart, their role in NHE1 regulation in this specific tissue has not been studied.

#### 3.2.6 Regulation by ERM Protein Family 

Ezrin, radixin and moesin, the ERM family form links between NHE1 and actin filaments of the cytoskeleton [120, 121]. This linkage helps facilitate cell migration [122]. NHE1 has ERM binding motifs in amino acids 552-560 of its cytoplasmic tail [121]. While not a great deal has been studied with regards to ERM proteins and the myocardium, one study [123] examined the intracellular location of ERM proteins in left ventricular cardiomyocytes and found that they were localized predominantly at the intercalated disc regions. With intracellular acidification this localization changed, with more localization of activated phospho-ERM in the transverse tubules, which is where NHE1 was localized. This effect was blocked with the NHE1 inhibitor cariporide. These results suggested that ERM proteins may mediate at least some of NHE1 activation in the myocardium.

#### 3.2.7 Regulation by Heat Shock Proteins 

Heat shock proteins, both Hsp70 and Hsp90, have been shown to be associated with NHE1 in several studies [101, 109, 110, 124] and inhibition of heat shock proteins can affect NHE1 activity [109, 125]. Hsp90 may affect NHE1 function through alteration of phosphorylation of the protein *via* AKT kinase [109]. A role of the association in inflammatory response has been suggested [126] and though the association has been shown in different tissues such as kidney and breast cancer cells [109, 110], it has not been extensively studied in the myocardium. One study has examined cardiofibroblasts [127] and showed the NHE1 interacted with Hsp70 by immunoprecipitation.

#### 3.2.8 Regulation by Carbonic Anhydrase II (CAII) 

Having an association with a membrane protein which moves ions such as those which CAII or other proteins produce, is thought to facilitate transport of the ions and is called a membrane transport metabolon [128]. CAII is a protein that catalyzes the hydration of carbon dioxide which leads to production of bicarbonate ions and protons. It occurs in a metabolon with NHE1 and other membrane ion transport proteins [128, 129]. A CAII-NHE1 interaction of this type has been studied in some detail in the myocardium. Initial studies characterized the fundamentals of the interaction and demonstrated that CAII binds to NHE1 *in vivo*, at the penultimate 13 amino acids of the regulatory cytosolic tail (Fig. 3). Ser796 and Asp797 form part of the CAII binding site on NHE1. The association of NHE1 and CAII was dependent on NHE1 being phosphorylated upstream of the CAII binding site [130, 131]. The association of these two proteins was then studied in the myocardium when varying myocardial stretch. Stretch is known to activate NHE1 (see Section 3.2.11) so the association of NHE1 with CAII was examined following stretch of rat papillary muscle by co-immunoprecipitation of the two proteins. Stretch increased association of NHE1 and CAII and inhibition of ${\mathrm{p90}}^{\text{RSK}}$ reduced the interaction, suggesting that phosphorylation was involved [132]. The same group [133] examined the association of NHE1 and CAII in obese type 2 diabetic mice. Both control (heterozygote) and obese mice showed co-immunoprecipitation of NHE1 and CAII, and they observed an increase in the amount of CAII attached to NHE1 in homozygote obese diabetic mice. It was suggested that there is an increase in the amount of this “membrane transport metabolon” in the failing mouse heart [133].

#### 3.2.9 Lipid Regulators of NHE1

Phosphatidylinositol 4, 5-bisphosphate is a different binding cofactor of NHE1, being a lipid and not a protein. It binds in two cationic juxtamembrane binding regions of NHE1, at amino acids 513-520 and 556–564 of the rat protein which are equal to amino acids 509-516 and 552-560 of human NHE1 [134]. Mutation of these binding sites decreases NHE1 transport efficiency [134]. The second region overlaps with a region between amino acids 542-598 which is called a lipid interacting domain, with a hydrophobic sequence ^573^LIAFY^577^ within it that binds the lipids diacyl glycerol and phorbol esters. These directly activate NHE1 [135]. Lipid regulators of NHE1 in the myocardium were examined some time ago, though not in the context of these lipid binding domains. Green *et al*. [136] showed that in cultured cardiac cells phorbol esters activate NHE and produce cellular alkalinization. Phorbol esters have also been shown to activate NHE1 in rat vascular myocytes [137] and Vigne *et al*. [138] showed that phorbol esters activate NHE in skeletal muscle myoblasts. Phorbol esters almost certainly act through these lipid binding sites since it has been shown that protein kinase C cannot directly phosphorylate the C-terminus of NHE1 directly [77] and NHE1 phosphorylation does not to correlate directly with protein kinase C activity [137].

#### 3.2.10 Role of ATP Binding 

NHE1 is an ATP binding protein. Early studies showed that depletion of intracellular ATP levels inhibits NHE1 activity [139, 140, 141, 142, 143]. More recently, direct binding of ATP to the NHE1 cytosolic domain was demonstrated by photoaffinity labeling and equilibrium dialysis. The location of ATP binding was localized to amino acids Gly542-Pro598 of human NHE1 (Fig. 3). ATP binding affected the pH dependence of NHE1 activity, ATP depletion caused an acidic shift in the ${\mathrm{pH}}_{\text{𝑖}}$ required for activation of NHE1 [144] which can shift the threshold for activation by about a half a pH unit [139, 145]. In cultured rat aortic smooth muscle cells, the activity of NHE1 has been shown to be reduced in response to hypoxic conditions with an increase in the threshold for activation of NHE1 [146]. ATP would be reduced under hypoxia which may account for this change in activity though direct ATP binding effects were not shown in this study [147]. A similar result was also demonstrated in cultured rat ventricular cells. Treatment of cells with 2-deoxyglucose demonstrated NHE-dependence on ATP levels [111]. Given that ischemia causes depletion of intracellular ATP levels, this study also suggests that NHE1 activity would be reduced during cardiac ischemia in the intact heart.

#### 3.2.11 Regulation of NHE1 by Stretch 

Stretch enhances myocardial contractility by two mechanisms. One rapid mechanism is the classic Frank-Starling mechanism that is attributed to enhanced myofilament calcium responsiveness. The second mechanism is the “slow force response” which occurs more slowly, as its name suggests. It is due to an increase in calcium transient size as a consequence of stretch activating autocrine and paracrine mechanisms [148, 149]. It is in the slow force response that NHE makes a significant contribution. Knockdown or inhibition of NHE1 blunts the slow force response [150, 151]. The mechanism by which this response works has been studied in several authors and was reviewed earlier [67, 148]. Briefly, stretch caused release of Angiotensin II, and activation of the AT1 receptor. This results in formation and release of endothelin which causes NHE1 hyperactivity. The increased NHE1 activity causes an increase in intracellular sodium and results in an increase in intracellular calcium though the reversal activity of the ${\mathrm{Na}}^{+}$${\mathrm{/Ca}}^{2+}$ exchanger. Concurrently elevated reactive oxygen species can trigger NHE1 phosphorylation. Some of this activation may be through the local hormones [148, 152]. Stretch activates angiotensin/endothelin dependent chain of events leading to increasing phosphorylation of Ser703 of NHE1 and elevated ERK phosphorylation [153]. Inhibition or knockdown of the mineralocorticoid receptor also reduces the activation by stretch, blocks reactive oxygen species elevation and blocks ERK and ${\mathrm{p90}}^{\text{RSK}}$ phosphorylation [152]. Additionally, knock down or blockade of the epidermal growth factor receptor also blocks this slow force response [153, 154]. Conversely, p38-MAPK activation after myocardial stretch limits ERK and ${\mathrm{p90}}^{\text{RSK}}$ phosphorylation, and NHE1 phosphorylation through activation of a dual specificity phosphatase which inhibits the slow force response [155]. This NHE1-dependent stretch induced slow force response is important since the increase in the calcium transient is thought to be involved in development of cardiac hypertrophy and therefore a contributor to heart failure. This mechanism possibly acts as an early step towards cardiac pathology if the stimulus remains over time. The activation of NHE1 after stretch may thus have important clinical implications (reviewed in [156]).

#### 3.2.12 Alteration in NHE1 Activity by Diet 

Relatively little research has been carried out to identify the impact diet has on NHE1 function and expression. Generally, high fat diets have been shown to increase oxidative stress and heart dysfunction. This may be associated with an activation of NHE1 because long term ablation of NHE1 activity *via* the use of NHE1-null mice mitigated the deleterious cardiac effects of the high fat diet [157]. However, perhaps the most powerful dietary intervention that demonstrated NHE-1 inhibition was provided by ginseng, a widely used medicinal herb particularly in Asian societies. Ginseng provided a direct anti-hypertrophic action in cultured cardiomyocytes, and importantly, inhibited heart failure through an attenuation of the upregulation of NHE1 activity typically exhibited in hypertrophic responses [158].

The mechanism whereby diet alters NHE1 activity is unclear. However, one may speculate that the membrane lipid composition which influences related membrane transporters like the ${\mathrm{Na}}^{+}$-${\mathrm{Ca}}^{2+}$ exchanger (NCX) [159, 160, 161, 162] may induce similar effects on NHE1 activity. In support of this hypothesis, acute administration to isolated cardiomyocytes of the omega-3 polyunsaturated fatty acids eicosapentaenoic acid (EPA) and docosahexaenoic acid (DHA) inhibited NHE1 activity [163]. Consistent with this effect, supplementing the diet with EPA and DHA in a rabbit model of volume and pressure overloaded cardiac hypertrophy and failure attenuated the upregulation in NHE1 activity [164].

### 3.3 SLC9A1 Gene Regulation

The NHE1 gene has been cloned from several species including the human, porcine and rabbit forms of the promoter [165, 166, 167]. The human gene has 12 exons and 11 introns and the mouse gene has a similar design [168]. There is a very large (41.5 kb) intron between the first and second exon while the other introns are much smaller [169]. Studies *in vitro *and in transgenic mice with the 5’ flanking region of the mouse promoter showed that NHE1 expression in the myocardium is high during early embryonic development and showed that the NHE1 protein is at relatively high levels in the neonate and declines with age [58, 59, 170, 171]. This effect may be comparable to the myosin heavy chain where a switch to fetal type of gene expression during hypertrophy increases expression.

A number of studies characterized transcription factors and regions important in expression in tissues outside the myocardium AP-1. The C/EBP family have been shown to be important in some cell types [172, 173]. The porcine, rabbit and mouse and human NHE1 promoter, are homologous particularly in the proximal 500 bp of the 5’ flanking region [165, 166, 167, 169]. Consensus sequences for the transcription factors AP-1, C/EBP and Sp1 are conserved between pig and human [167]. A proximal AP-2 binding site in the mouse NHE1 promoter is important in expression in fibroblasts and in P19 embryonal carcinoma cells [57, 165]. The NHE1 promoter is activated in some models of cell differentiation including in P19 cells or in L6 muscle cells [174, 175]. Some other regions of the promoter important in expression are a poly (dA:dT) region of the NHE1 promoter is located at bp -155 to -169 of the mouse gene which was tested in L6 and NIH 3T3 cells [176]. Several other regions of the NHE1 promoter were studied in a variety of cells. COUP-TF (Chicken ovalbumin upstream promoter transcription factor) type I and II is more distal (-841 to -800 bp) and is also important in expression [177]. Thyroid hormone receptor TRα_1_ is also implicated in regulation of the promoter [178].

Studies directly examining NHE1 promoter regulation in the myocardium are rare. A 1.1 kb region of the mouse promoter drove expression in cardiomyocytes that was stimulated by serum. Deletion of a proximal AP-2 site decreased promoter activity 4-fold [179]. Mutation of that site, combined with deletion of distal regions of the promoter, virtually eliminated promoter activity in cardiomyocytes. DNase I footprinting analysis showed that the poly(dA:dT) rich region (-155 to -169), is protected by heart nuclear extracts as is the COUP-TF element [176, 180]. Thyroid hormone may regulate the NHE1 gene in the heart. Treatment of heart cells with thyroid hormone increased protein binding of nuclear extracts in the COUP-TF region and treatment of cardiomyocytes with thyroid hormone increases NHE1 protein expression [178].

NHE1 levels have been shown to be elevated in the myocardium following ischemic heart disease and some of this could be mediated through reactive oxygen species (ROS). Increasing serum from 0.5 to 10% induces NHE1 promoter activity in NIH3T3 fibroblasts [179]. The increase correlates with increased $O_{2}$ superoxide production and NHE1 promoter activity and $O_{2}$ superoxide production could be blocked by the oxidase inhibitor diphenyleniodonium [181]. Tiron, a specific $O_{2}$ superoxide scavenger, could also block increases in NHE1 promoter activity and protein levels [181].

Aside from these earlier studies, there has been a surprising dearth of recent work on the promoter in the myocardium. It would be interesting to examine NHE1 transcription in detail during cardiac hypertrophy and in response to ischemia and reperfusion.

## 4. Structure of NHE1 and Location of Inhibitor Binding Site 

**Fig. 4.**
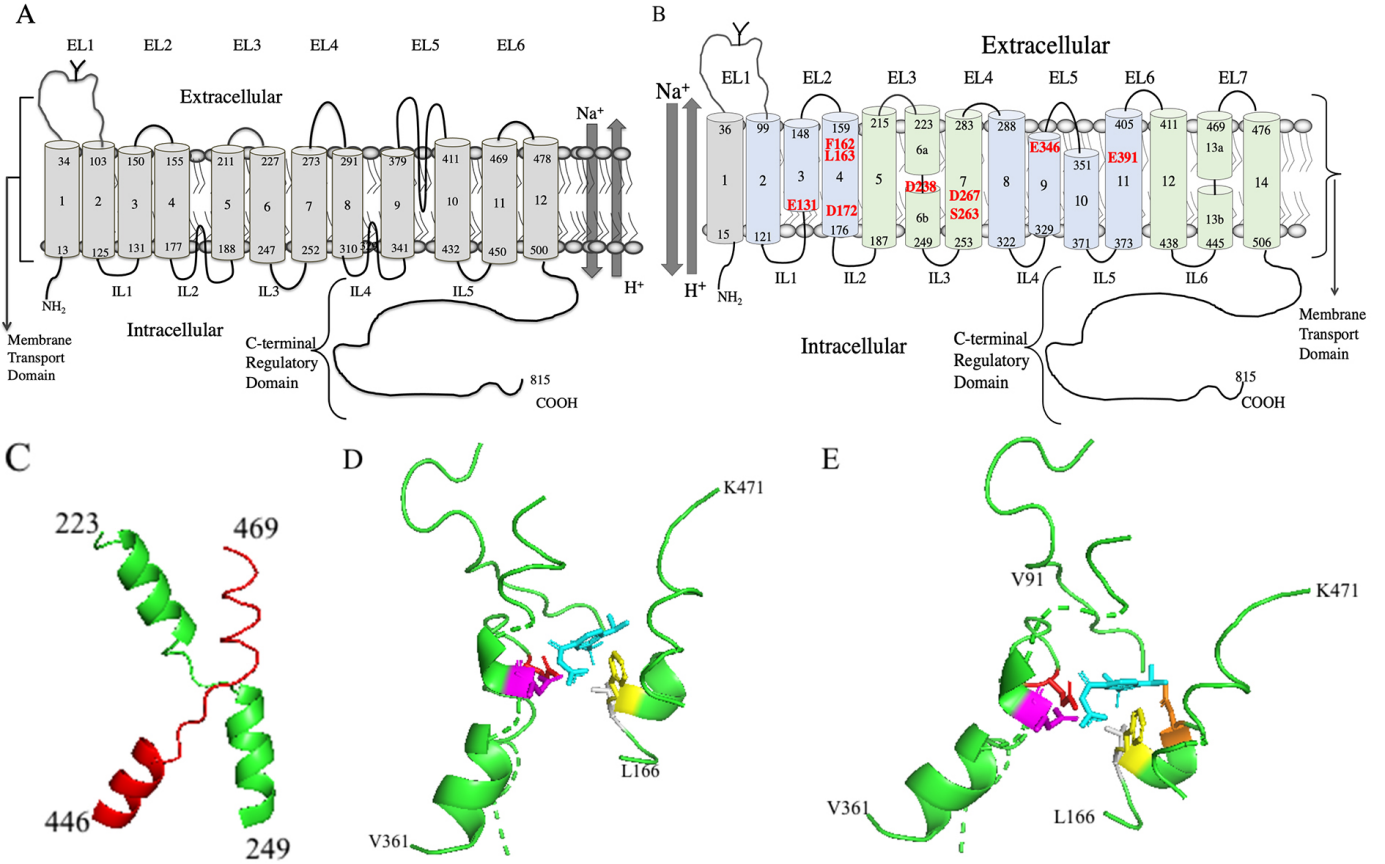
** Molecular models of human NHE1**. (A) Two-dimensional topology model of human NHE1 based on hydrophobicity analysis and cysteine accessibility studies [182, 183]. (B) Novel two-dimensional topology model of human NHE1. TM’s 2-14 are based on the Cryo-EM structure [11]. TM1 is added as per [182, 183, 184]. The core domain TMs are colored light green and the dimerization domains TMs are light blue. Amino acid numbering indicates the end of the transmembrane segments. EL, extracellular loop; IL, intracellular loop. The transmembrane segment number is indicated beginning from the N-terminus and including the first TM. The unwound region of TMs 6 and 13 is indicated by a discontinuity. Amino acids important in cation coordination, proton sensing or inhibitor binding are indicated in red. (C) Structure of human NHE1 fold from [11]. TM 6 (green) and 13 (red) of human NHE1 are shown (amino acids 223-249 and 445-469 respectively). (D) Lateral view of cariporide binding site in the Cryo-EM structure [11] of human NHE1. Most of the protein is not shown. Cariporide cyan, E346 red, D267 magenta, F162 yellow, L163 grey. (E) Lateral/extracellular view, color scheme retained as in D, plus D159 orange.

The structure of NHE1 and the inhibitor binding site is of paramount interest as clearly the binding site will affect the efficacy and potency of inhibitors and the site itself will affect the design of novel inhibitors. Traditionally, human NHE1 has been thought of as a 12 transmembrane protein with a large cytoplasmic tail (Fig. 4A, Ref. [182, 183, 184]). This type of low-resolution model was initially based on hydrophobicity plots and later more experimental evidence was added, mostly using cysteine accessibility studies along with hydrophobicity plot [182, 183]. These studies initially suggested 12 transmembrane segments were present with two intracellular re-entrant loops, one between TM4 and TM5 and one between TM8 and TM9. There was a larger extracellular loop predicted between TM9 and T10.

The structure of the *E. coli* NhaA (${\mathrm{Na}}^{+}$${\mathrm{/H}}^{+}$ antiporter type A) protein was deduced in 2005 [185] and this led to attempts to model human NHE1 based on the structure of the *E. coli *protein [186]. However, this led to some conflicts in interpretation [183, 187, 188]. The *E. coli* protein, NhaA (for ${\mathrm{Na}}^{+}$${\mathrm{/H}}^{+}$ antiporter type A) has significant differences from the mammalian protein. For example, the stoichiometry of exchange in bacteria is ${\mathrm{1Na}}^{+}$${\mathrm{/2H}}^{+}$ [189] compared to the electroneutral 1:1 exchange of mammalian NHE1. However, it is worth briefly reviewing a few fundamental aspects of this structure which are similar in other forms of this type of transport (see also reviews [190, 191, 192]). The crystal structure of acid-inactivated NhaA has 12 TM segments. A critical feature is a TMIV-XI assembly. Both these helices are discontinuous, interrupted by an extended segment. The discontinuous helices form mid-membrane dipoles. It was suggested that the charge on D133 compensates for the opposing N-terminal end and K300 for the opposing C-terminal end of the helix [185, 191]. The pore contains cytoplasmic and periplasmic funnels, both of which narrow so that hydrated cations cannot pass. The ion binding site of NhaA is formed around the extended segments of TMs IV and XI and includes D164, D163, and T132 [193, 194]. It is hypothesized that ${\mathrm{Na}}^{+}$ binding to the active site from the cytoplasm causes a charge imbalance. This triggers movements of the TMIV-XI assembly, exposure of ${\mathrm{Na}}^{+}$ to the periplasm, its release, and proton. Although this model for the function of NhaA is well developed and elegant, there is some controversy. Arkin *et al*. [195] proposed a variation of the model, in which 3 conserved Asp residues are key to ${\mathrm{Na}}^{+}$${\mathrm{/H}}^{+}$ antiport. D164 is the ${\mathrm{Na}}^{+}$ binding site, D163 controls accessibility, and D133 mediates pH regulation. More recently, K300 was proposed to be essential for stability but not for electrogenic transport [196].

Structures of other ${\mathrm{Na}}^{+}$${\mathrm{/H}}^{+}$ exchangers have been more recently elucidated. This includes that of the archaeal ${\mathrm{Na}}^{+}$${\mathrm{/H}}^{+}$ antiporter NhaP1 from *Methanococcus jannaschii * [197, 198], NapA from *Thermus thermophilus * [199, 200], NhaP [201] (a ${\mathrm{Na}}^{+}$/H${\mathrm{/H}}^{+}$ exchanger of *Pyrococcus abyssi*, an archaeon from deep-sea hydrothermal vents), NHE9 [202] (another mammalian isoform related to NHE1), NHA2 [203] (a more distant mammalian ${\mathrm{Na}}^{+}$${\mathrm{/H}}^{+}$ exchanger more closely related to NhaA). Generally, a summary would be to that these ${\mathrm{Na}}^{+}$${\mathrm{/H}}^{+}$ exchanger types have significant similarities to NhaA of *E. coli* despite their low homology to the *E. coli* protein. Functionally this includes acid residues for cation coordination, and the presence of a ${\mathrm{Na}}^{+}$${\mathrm{/H}}^{+}$ exchanger fold consisting of discontinuous transmembrane segments with a mid-membrane unwound stretch of amino acids. Some publications have also suggested alternating access to key acidic (Asp) residues using elevator like structural movements of transporter domains [193, 200, 202].

Very recently, the structure of human NHE1 in complex with the regulatory protein CHP1, was determined by cryogenic electron microscopy (Cryo EM) [11]. The structure was of amino acids 87-590, which formed 13 transmembrane helices and 3 cytosolic helices. The topology was generally consistent with previous models except a previous re-entrant loop between a previously labeled TM9 and TM10, is actually two TM helices. A simplified model of the topology is shown in Fig. 4B. The model is based on the Cryo EM structure of human NHE1. TM 1 was added based on an earlier molecular model of NHE1 and experiments involving cysteine labeling of TM 1 which showed that TM 1 was present in the intact protein [182, 183, 184] (Numbering of NHE1 TMs will from here on include TM1 as shown in Fig. 4B.)

NHE1 was a homodimer in the Cryo EM structure. Each monomer was made of a dimerization domain of TM segments (TMs 2-4, and 8-11, amino acids 99-176, plus 288-405) plus a core domain (TMs 5-7 and 12-14, amino acids 187-283 and 411-505) [11] (see Fig. 4B). TMs 6 and 13 (amino acids 223-253 and 445-469) have the NHE-fold with an unwound region in the middle and they cross each other (Fig. 4C). The helix breaks are thought to participate in forming the ion permeation pathway. In their structure there was a funnel between the dimerization domain and the core domain of each protomer. The funnel is formed by TMs 2,3,4,6,7 and 11. Proton-titratable residues in the funnel are E131, D172, D238, D267 and E391 (indicated in Fig. 4B). This results in a negatively charged cavity which was thought to participate in cation binding and proton sensing [11]. D267 was thought to participate in cation binding and is critical to activity [204, 205]. The S263 sidechain was also thought to participate in ion transport and D238 was thought to indirectly participate, coordinating a water molecule [11]. E391 is essential [204] but because of its location away from the cation binding site it may affect folding or stability of the protein and was not thought to directly binding cations. E131 was thought to be a pH sensor functioning when protonated to accelerate cation release [11]. Mutation of F162 has been shown to have a large effect, reducing efficacy of cariporide and mutation of I169 and I170 accentuate this effect [206]. Mutation of E346 has also been shown to have large effects on inhibitor efficacy [207, 208].

Considering the potential benefits of NHE1 inhibition in cardiac disease, there has always been great interest in the determination of the exact site of inhibitor binding. Classically, a number of studies carried out site specific mutagenesis on amino acids believed to be involved in inhibitor binding. Then any changes in inhibitor efficacy were examined. Some of these studies have suggested a number of amino acids with large effects on inhibitor potency as being important in human NHE1 inhibition that includes human F167 [209], L167 (rat) [208], Gly174, Leu163 [210] while other studies have showed smaller changes in drug potency with mutation of human L265 and L255 [211]. Other studies [208, 212, 213] used large scale replacement of pieces of NHE1 to try to determine which regions are critical in drug interaction, but these have the disadvantage of increased likelihood of disruption of the structure of NHE1. Molecular modeling of NHE1 and inhibitor docking was also attempted [184] and a number of amino acids were suggested to interact with inhibitors at different potential binding sites.

While these studies provided interesting information, the recent Cryo-EM structure directly showed the inhibitor binding site of the benzoylguanidine cariporide [11]. Cariporide bound from the extracellular side of NHE1 in a pocket located between the dimerization and core domain. It was surrounded by TMs, 4, 7, 9, 10 and 13. The extracellular loop #1 from the opposing subunit, and resides of TM 9 and 10 may be essential for the binding pocket. Amino acid D267 of TM7 points up into the inhibitor binding pocket and interacts with the positively charged group of the guanidine (Fig. 4D,E) [11]. The side chain of F162 (TM4) also interacts with the guanidine group and the phenyl ring of cariporide which agrees with mutational studies [206]. The guanidine group is also coordinated by the side chain of E346 (TM9) which agrees with another mutational studies [207]. Cariporide also has a methylsulfonyl group at the meta- and para- position of the phenyl ring (Fig. 2B). These are buried in a sub pocket formed by D159, L163, D95, H98 and V99. Mutational studies, including of L163, support the importance of these amino acids in inhibitor binding [209].

It is also important to note that it has long been suggested that inhibitors such as amiloride, bind to the same or are overlapping with, the cation binding site (reviewed in [214]). The Cryo-EM structure of NHE1 could not visualize the precise location of the ${\mathrm{Na}}^{+}$ ion, which is thought to be partially hydrated, and is about the same size of the guanidine group [11, 214]. Given the coordinating amino acids, their characteristic charges, location, and the size of the pocket, the authors hypothesized that F162, D267 and E346 coordinate the ${\mathrm{Na}}^{+}$ ion in the outward facing site [11]. Mutation of these residues affects both inhibitor efficacy and activity, supporting this hypothesis [215].

## 5. Development of Inhibitors of NHE1: Past and Present

Because of the extensive experimental evidence that NHE1 participates in cardiac pathologies including ischemia and reperfusion injury as well as myocardial remodeling and heart failure (discussed in Sections 6 and 7) there has been substantial interest in inhibition of NHE1 and in the development of clinically useful compounds for treatment of the diseased myocardium. Early pharmacological studies probing the function of NHE1 in biological systems including heart disease employed the use of amiloride (Fig. 2A), a potassium-sparing diuretic or its derivatives such as ethylisopropylamiloride (EIPA), methylisobutyl amiloride (MIA), hexamethyl amiloride (HMA), dimethylamiloride (DMA) (Fig. 2C) and others. While these agents are effective in inhibiting NHE1, they lack specificity against the NHE1 isoform, have non-specific effects on many aspects of cardiac performance [216] and are also effective against other ion regulatory systems. This concern regarding non-specificity was rectified to a large degree by the development of novel second generation NHE-1 specific inhibitors by the pharmaceutical industry. The first among these was the benzoylguanidine derivative HOE694 ((3-methylsulphonyl-4-piperidino-benzoyl) guanidine methanesulphonate developed by Hoechst AG (now part of Sanofi) and which was demonstrated to exert cardioprotective and antiarrhythmic effects in ischemic and reperfused hearts [32]. This was followed by the development of a new NHE1-specific inhibitor initially designated as HOE642 (N-(Aminoiminomethyl)-4-(1-methylethyl)-3-(methylsulfonyl)-benzamide) [31] and subsequently renamed as cariporide (Fig. 2B) in preparation for clinical development. As will be evident from the discussion below, cariporide is the most extensively studied of the NHE1-specific inhibitors both experimentally as well as in clinical studies particularly with respect to mitigating ischemic and reperfusion injury.

The development of these benzoyl guanidine derivatives has led to the rapid formulation of numerous newer and more potent NHE1-specific inhibitors. For an in-depth description of the development and chemistry of NHE1-specific inhibitors please refer to [217]. A partial list of these agents is provided in Table 1 (Ref. [32, 218, 219, 220, 221, 222, 223, 224, 225]). Virtually all of the agents listed in this table possess the monocyclic acylguanidine structure found in the amiloride-based inhibitors (with the exception of SL 59.1227) but demonstrate markedly enhanced specificity towards NHE1 as well as greater potency. Preclinical and clinical studies with a number of these agents are discussed in greater detail below.

**Table 1.** **Some examples of NHE1-specific inhibitors developed by the pharmaceutical industry**.

Drug	Chemical name	Developer	Reference
HOE694	(3-methylsulphonyl-4- piperidino -benzoyl) guanidine methanesulphonate	Hoechst ${\mathrm{AG}}^{4}$	[32]
${\mathrm{HOE642}}^{1}$	(N-(Aminoiminomethyl)-4-(1-methylethyl)-3-(methylsulfonyl) -benzamide)	Hoechst AG	[218]
MS31-038	2-phenyl-8-(2-methoxyethoxy)-4-quinolyl carbonylguanidine bismethanesulfonate	Mitsui	[219]
EMD875802	N-carbamimidoyl-2-methyl-4,5-bis(methylsulfonyl) benzamide	Merck ${\mathrm{KGaA}}^{5}$	[220]
EMD851313	(2-Methyl-5-(methylsulfonyl)benzoyl)guanidine	Merck KGaA	[221]
T162559	((5E,7S)-[7-(5-fluoro-2-methylphenyl)-4-methyl-7,8-dihydro-5(6H)-quinolinylideneamino] guanidine dimethanesulfonate)	Takeda	[222]
BIX	N-[4-(1-acetyl-piperidin-4-yl)-3-trifluoromethyl-benzoyl]-guanidine	${\mathrm{BI}}^{6}$	[223]
SL59.1227	3-[(cyclopropylcarbonyl)amino]-N-[2-(dimethylamino)ethyl]-4-[4-(5-methyl-1H-imidazol-4-yl)piperidin-1-yl]benzamide	Sanofi	[224]
Zoniporide	[1-(quinolin-5-yl)-5-cyclopropyl-1H-pyrazole-4-carbonyl] guanidine	Pfizer	[225]

^1^ cariporide; ^2^ rimeporide; ^3^ eniporide; ^4^ currently a wholly-owned subsidiary of Sanofi; ^5^ now Merck Serono (EMD Serono in the United States and Canada);^6^ Boehringer Ingelheim.

Recently one group has developed a novel series of compounds of 6-substituted amiloride and hexamethylene amiloride (Fig. 2C) derivatives as inhibitors of NHE1. Depending on the precise compound made, they may also inhibit human urokinase plasminogen activator [226, 227, 228]. The reasoning behind modification of amiloride (and its derivative) for use as an inhibitor is that amiloride is a pyrazinoyl guanidine, a clinically safe compound used as a potassium-sparing diuretic. Given the problems the benzoyl guanidine derivative cariporide for treatment of heart failure (see below) it was reasoned that using a pyrazinoyl guanidine core that differs in the aromatic core from cariporide (a benzoyl guanidine, Fig. 2) and related NHE1 inhibitors might be a prudent approach to avoid side effects and regulatory issues for drug development. 6-substituted amiloride/HMA compounds have increased potency towards NHE1. Amiloride is an ideal candidate for a scaffold to build a dual-targeting compound with cardiovascular beneficial properties. HMA is both significantly more potent and more specific towards NHE1; amiloride inhibits sodium channels but HMA much less so [229]. Inhibitors have been developed with nM potency towards NHE1 and show no effects on diuresis or urinary ${\mathrm{Na}}^{+}$${\mathrm{/K}}^{+}$ level in rats [226, 227, 230]. While in theory these compounds sound extremely promising, they have not yet been tested for efficacy in prevention of ischemic and reperfusion injury or in the prevention of deleterious cardiac remodeling.

It is of note that some of these compounds have a dual action, inhibiting urokinase plasminogen activator (uPA) [226, 227, 230]. In the myocardium, uPA induces cardiac fibrosis and human hearts with end stage failure and fibrosis have elevated plasminogen activator activity [231]. Excess uPA promotes cardiac fibrosis in association with M2 macrophages [232]. Blocking the uPA pathway reduces cardiac macrophage accumulation, excess collagen formation and heart fibrosis [233]. Thus, it may also be of interest to test the effect of dual inhibition of NHE1 and uPA, in heart failure models. To our knowledge this has not been done. Caution is advised though, as urokinase inhibition could increase thrombolysis and thrombosis. Fortunately, a large number of NHE1 inhibitory compounds have been made with varying degrees of uPA inhibition from none, to little, to potent inhibitors of both uPA and NHE1 [226, 227, 230].

## 6. Role of NHE1 in Cardiac Ischemic and Reperfusion-Induced Injury

### 6.1 Theoretical Concepts

**Fig. 5.**
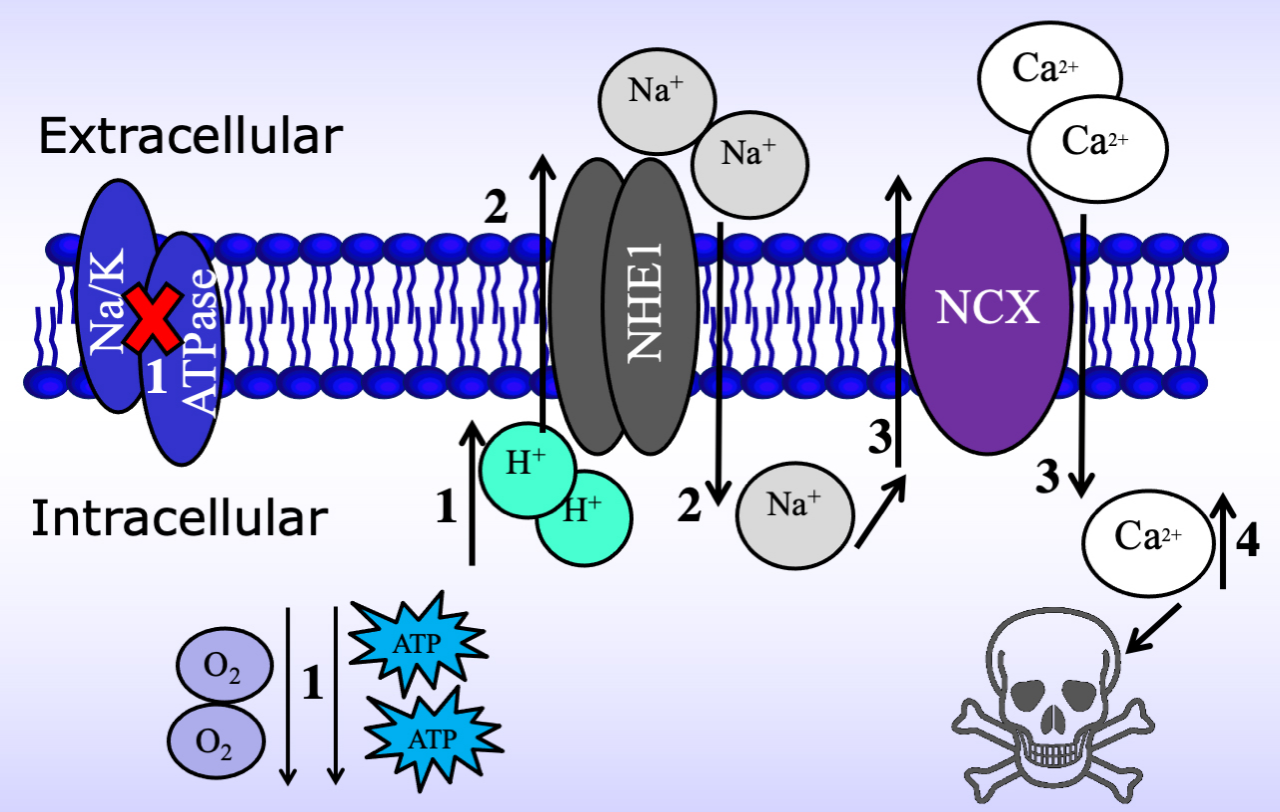
**Schematic of the mechanism by which the ${\mathrm{Na}}^{+}$${\mathrm{/H}}^{+}$ exchanger (NHE1) and the ${\mathrm{Na}}^{+}$${\mathrm{/Ca}}^{2+}$ exchanger (NCX) interact to generate cardiac injury and dysfunction during ischemia/reperfusion challenge**. (1) In the presence of reduced coronary blood flow and absence or reduced oxygen, ischemia causes a reduction in intracellular ATP levels and an accumulation of intracellular $H^{+}$. The decreased levels of ATP stores impair ${\mathrm{Na}}^{+}$${\mathrm{/K}}^{+}$ ATPase activity reducing ${\mathrm{Na}}^{+}$ export. (2) The elevated intracellular $H^{+}$ activates NHE1 to remove the $H^{+}$ in exchange for extracellular ${\mathrm{Na}}^{+}$. (3) The increasing intracellular ${\mathrm{Na}}^{+}$ via NHE1, slows the removal of intracellular calcium by NCX or drives NCX in the reverse direction thereby increasing intracellular calcium concentrations in the cell. (4) The excess entry of ${\mathrm{Ca}}^{2+}$ in the cell results in contractile dysfunction and structural damage to the myocardial cell.

Although the entry of excessive extracellular ${\mathrm{Ca}}^{2+}$ into the cardiomyocyte was recognized early on as a key event in the toxic effects of ischemic injury to the heart (${\mathrm{Ca}}^{2+}$ overload) [234], the mechanism whereby this excess ${\mathrm{Ca}}^{2+}$ entered the cell remained unknown until decades later. Blocking the L-type ${\mathrm{Ca}}^{2+}$ channel had limited clinical utility in protecting the heart from ischemic and reperfusion injury so that was clearly not the mechanism for ${\mathrm{Ca}}^{2+}$ entry [235]. Lazdunski proposed in 1985 [24] that a mechanism involving NHE and NCX activation was responsible for the toxic entry of ${\mathrm{Ca}}^{2+}$ into the myocardium as is summarized in Fig. 5. This scheme reinforces the concept of a close link between NHE1 and NCX not only with regards to ischemia and reperfusion but also pertaining to NHE1-dependent cardiac hypertrophy, as discussed in section 7 of this review. However, NHE1 inhibition has been shown to attenuate the deleterious effects of many factors including to varying degrees, interactions with G-protein-coupled receptors [236], the α_1A_ adrenoceptor subtype [75], protein kinases [84, 87, 237], angiotensin I and II receptors [69], membrane lipids [238] and endothelin-1 [239]. The primary cardioprotective action of the inhibitors, however, remains in their ability to block ${\mathrm{Na}}^{+}$, $H^{+}$ and ${\mathrm{Ca}}^{2+}$ movements *via* the NHE1 and NCX transport pathways in the myocardial cell (Fig. 5).

### 6.2 Studies with Amiloride Analogues

Karmazyn’s laboratory was the first to demonstrate the likely validity of this concept by demonstrating a cardioprotective effect of amiloride, in isolated perfused rat hearts subjected to ischemia and reperfusion [240]. This seminal observation using a well-known inhibitor of ${\mathrm{Na}}^{+}$ transport (amiloride) was independently confirmed and extended by two other labs shortly thereafter. Tani and Neely demonstrated both metabolic and ionic data consistent with an involvement of the NHE and NCX pathways in ischemic/reperfusion injury [241]. By this time, Edwin Cragoe, Jr. had established a large library of amiloride analogues at the Merck laboratories [242] that were more specific inhibitors of the NHE pathway than amiloride and this significantly accelerated the work in this area. Several laboratories took advantage of this advancement and employed several of these amiloride analogues in a series of studies to again elicit significant cardioprotection during ischemia and reperfusion challenge to the heart [243, 244, 245, 246, 247].

A biochemical approach further indirectly implicated the NHE pathway in the cardiac damage by modifying the extracellular acidity and ${\mathrm{Na}}^{+}$ levels during the ischemia and reperfusion insult [243, 245, 248, 249]. Ionic changes consistent with a role for NHE during ischemia/reperfusion and its blockage by NHE inhibitors were demonstrated by radioisotopic [241], atomic absorption spectrometric [245], electrophysiological [248] and nuclear magnetic resonance methods [250]. The cardioprotection was observed whether the NHE inhibitors were delivered prior to ischemic insult or solely during reperfusion although protection was generally greater when the drug was present during the ischemic period [251, 252]. The cardioprotective effects of NHE blockers were not species specific. Protection was observed in hearts from rats and guinea pigs [247].

All of these studies were consistent with a critical involvement of NHE in the cardiac damage [240, 241, 253, 254], cardiac contractile dysfunction [240, 241, 243], and arrhythmias [255, 256] that are evident during the ischemia and reperfusion insult. NHE inhibitors were cardioprotective not only during ischemia and reperfusion challenge but protected the myocardium during hypoxia/reoxygenation insult [257], hypothermic conditions [258, 259], oxidative stress [260, 261], and pacing induced-heart failure [262]. Conversely, NHE inhibitors did not provide cardioprotection during ischemic pre-conditioning [259] or the ${\mathrm{Ca}}^{2+}$ paradox protocol of myocardial ${\mathrm{Ca}}^{2+}$ overload [257].

### 6.3 Studies with New Generation NHE1 Specific Inhibitors 

Earlier studies using amiloride or its analogues as cardioprotective agents presented some difficulty in interpretation as they lacked specificity in terms of targeting NHE1. Moreover, virtually all studies with these agents were carried out using *in vitro* cardiac preparations with no studies on more clinically-relevant *in vivo* animal models. This situation was rectified to large degree by the development of highly specific NHE1 inhibitors as discussed in section 5. The first of these agents to be developed was the benzoyl guanidine derivative HOE 694 which was shown to produce extensive protection in both isolated rat hearts as well as in rats subjected to coronary artery ligation [32]. Although HOE 694 was not destined for clinical development this was not the case for HOE 642 (cariporide) (Fig. 2B), a more potent NHE1 inhibitor, developed shortly thereafter [218] and which, in an initial study was shown to exert potent cardioprotective effects in both isolated hearts as well as *in vivo* coronary artery ligation followed by reperfusion [31]. This protection was manifested in terms of reduction in the incidence of arrhythmias, reduction in tissue injury as well as preservation of energy metabolites [31]. Cariporide subsequently emerged as the most widely studied of the newer generation of NHE1 inhibitors as a cardioprotective strategy. Indeed, these studies using cariporide have shown excellent cardioprotection across different animal species and experimental models [253, 254, 263, 264, 265, 266, 267].

The consistent protection seen with cariporide was clearly evident in subsequent studies utilizing newer NHE1 specific inhibitors including AVE 4890 [268], MS-31-038 [219], BIX [223], BIIB 513 [269], EMD 85131 [221] and others. Indeed, it can be safely stated that virtually all animal studies using these agents have consistently demonstrated excellent cardioprotection irrespective of experimental model or animal species. This consistency in demonstrating cardioprotective efficacy of NHE1 inhibitors is likely unmatched by any other strategy. Indeed, Garrett Gross’ laboratory at the Medical College of Wisconsin has reported that NHE1 inhibition with BIIB 513 was superior to ischemic preconditioning particularly when ischemic preconditioning was no longer effective as a result of prolonged ischemic duration [221].

### 6.4 Studies with NHE1 Transgenic Mice

A number of studies were undertaken to determine whether genetic modulation of NHE1 alters the cardiac response to ischemia and reperfusion. Surprisingly, overexpression of NHE1 in transgenic mouse hearts resulted in enhanced recovery after reperfusion of ischemic isolated perfused hearts which the authors attributed to improved metabolic parameters [270, 271]. Ironically, in one study the protection seen with NHE1 overexpression was similarly observed with cariporide [271]. In another study, cardiac overexpression of NHE1 resulted in modest protection against ischemia and reperfusion *in vivo* although this was not affected by zoniporide, a highly specific NHE1 inhibitor suggesting that the protection was not NHE1 dependent [272]. With aging, NHE1 overexpressing mice exhibited increased apoptosis, left ventricular contractile dysfunction, myocardial remodeling and premature death which the authors attributed to sustained endoplasmic reticulum stress [272]. While further work is required in this area, when taken together the results suggest that NHE1 overexpression *per se* may not be necessarily deleterious to the ischemic myocardium although inhibiting the exchanger to a critical level confers protection. This concept is reinforced by the finding that genetic ablation of NHE1 in mice confers protection against ischemic and reperfusion injury in isolated perfused hearts [273].

### 6.5 NHE1 Inhibition for Post-Cardiac Arrest Resuscitation: An Ischemia and Reperfusion Scenario

Successful cardiac resuscitation, particularly after out-of-hospital sudden cardiac arrest, represents a major medical challenge. This reflects the very poor outcome in terms of successful resuscitation which is seen in only about 10% or less of cardiac arrest victims, although these rates vary depending on various factors [274]. Cardiac resuscitation is in essence an ischemia (cardiac arrest) and reperfusion (resuscitation) scenario thus suggesting a potential benefit of cardioprotective agents in terms of improving resuscitation efforts. Indeed, NHE1 inhibitors have been proven to be of immense benefit for improving post-cardiac arrest resuscitation in experimental animal models. Much of the original and pioneering work in this area comes from Gazmuri’s laboratory in Chicago who first proposed this concept (see below) and who recently published a comprehensive review of the role of NHE1 in cardiac resuscitation [275]. As such only a brief discussion is presented here.

As just noted, the first report demonstrating a beneficial effect of NHE1 inhibition was presented by Gazmuri and colleagues who showed that cariporide improved post ventricular fibrillation recovery in isolated rat hearts as well as rats *in vivo* subjected to cardiac arrest [276]. It is interesting that with respect to the latter, cariporide reduced the degree of chest compression required to attain sinus rhythm and precluded the necessity for electrical defibrillation in six of eight animals studied whereas electrical defibrillation was required in all 8 control rats [276]. Moreover, and importantly, over 90% of cariporide treated animals survived the cardiac arrest-resuscitation protocol compared to just over 60% seen in control animals [276]. A similar beneficial effect of cariporide was also convincingly demonstrated by these and other investigators using a pig model of cardiac arrest followed by attempted resuscitation [277, 278]. Thus, cariporide improved hemodynamics following resuscitation compared to control animals, completely prevented mortality while reducing neurological deficit [278].

From a mechanistic perspective, the beneficial effects of NHE1 inhibition in improving cardiac resuscitation appear to be similar to mechanisms seen in classic cardioprotection seen in ischemic and reperfused hearts with NHE1 inhibitors. Thus, it has been reported that the NHE1 specific inhibitor AVE4454B, although less effective than cariporide in terms of improving cardiac resuscitation [279], improved cardiac resuscitation in the rat which was associated with diminished cardiac mitochondrial calcium overload [280]. The beneficial effects of cariporide were also associated with diminished arrhythmogenesis including ectopic activity and reduced the shortening of the action potential duration associated with resuscitation [277, 281]. Further mechanistic insights into the improved resuscitation produced by NHE1 inhibitors were provided in a study using the NHE1 specific inhibitor zoniporide. In this regard zoniporide, as expected, improved ventricular recovery following ventricular fibrillation-induced cardiac arrest in pigs, effects associated with improved myocardial metabolic status including preserved myocardial creatine phosphate to creatine ratios thus indicating improved oxidative phosphorylation [282]. Mitochondrial as well as neuronal protection with cariporide was also demonstrated in an asphyxia model of cardiac arrest in the rat although the protection was evidently dependent on cariporide dose [283]. Thus, while a protective response was seen using either 1 or 3 mg/kg cariporide no protection was evident when the cariporide dose was increased to 5 mg/kg [283]. The protective effect of the two lower cariporide doses was enhanced under hypothermic resuscitation conditions whereas no additional benefit was observed with 5 mg/kg cariporide [283]. Taken together, the latter results suggest that the optimal beneficial effect of NHE1 inhibitors in cardiac resuscitation may be dependent on various factors including drug dose and resuscitation conditions.

Finally, it should be added that a contribution to the beneficial effect of cariporide in cardiac resuscitation may reflect improved hemodynamics, particularly in conjunction with the initial chest compression. Thus, Gazmuri’s group has shown, using a rat closed chest ventricular fibrillation model, that cariporide enhances hemodynamic efficacy of resuscitation by producing comparable or higher systemic as well as regional blood flows while at the same time reducing depth of compression, a finding of substantial clinical relevance in terms of improving efficacy of closed chest cardiopulmonary resuscitation [284].

## 7. Role of NHE1 in Cardiac Hypertrophy, Remodeling and Development of Heart Failure

### 7.1 Sodium as a Key Factor in the Hypertrophic Program 

The role of sodium in the development of cardiac hypertrophy is well established. High dietary sodium is associated with an increased incidence of cardiac hypertrophy and heart failure. Indeed, heart failure patients exhibit defective sodium cellular regulation which could compound the deleterious effects of sodium on cardiac pathology [285]. Although one of the mechanisms associated with sodium-induced cardiac hypertrophy involves the secondary response to chronic hypertension, substantial evidence supports a direct effect of sodium on myocardial hypertrophy thus directly contributing to myocardial remodeling and the development of heart failure. Thus, elevations in sodium concentrations directly produce hypertrophy in cultured myocardial myoblasts [286] and administering a high sodium diet to rats produces cardiac hypertrophy independently of blood pressure elevation [287]. Moreover, the sodium channel blocker tetrodotoxin prevents isoproterenol-induced hypertrophy in cultured H9c2 cardiomyoblasts [288]. One of the multiple sodium-dependent direct mechanisms proposed to induce cardiac hypertrophy involves the elevation of intracellular calcium concentrations due to reduced calcium efflux *via* the NCX or reverse mode NCX activity [289]. This mechanism is supported by studies showing that selective chronic inhibition of NCX inhibits cardiac hypertrophy in nephrectomized hypertensive rats, a model of heart failure with preserved ejection fraction, independently of blood pressure reduction [290]. However, elevations in intracellular sodium concentrations can directly produce hypertrophy by stimulation of intracellular signaling molecules linked to the hypertrophic program, including protein kinase C [291] as well as reactive oxygen species [292]. Moreover, high intracellular sodium concentrations may also contribute to cardiac hypertrophy by altering mitochondrial dynamics resulting in metabolic remodeling although the precise mechanisms underlying these events have not been fully elucidated [293]. As discussed below, studies using NHE1-specific inhibitors have demonstrated a multitude of intracellular mechanisms potentially contributing to a sodium dependent hypertrophic program. It is interesting to point out that NHE-dependent cardiac hypertrophy manifests primarily as pathological hypertrophy and does not seem to be involved in physiological adaptive hypertrophic responses. In this regard, it has been proposed that AKT (protein kinase B)-dependent NHE1 phosphorylation prevents NHE1 overactivation in physiological hypertrophy [294] and thus it is likely that NHE1 activation does not partake in the physiological hypertrophic program. High intake of dietary sodium in experimental animals has also been linked to activation of the adrenergic system, particularly that involving α1 receptor activation resulting in a myocardial hypertrophic response [295]. The critical role of sodium in the development of cardiac hypertrophy has been shown not only in experimental animals but also clinically in studies demonstrating that high urinary sodium excretion is independently associated with the development of left ventricular hypertrophy in both non-diabetic hypertensive subjects [296] as well as in patients with Type 2 diabetes [297].

### 7.2 Studies with NHE1 Inhibitors

A number of studies have documented an important role of NHE1 in mediating the enhanced intracellular sodium elevations, particularly under ischemic insult. For example, Bak and Ingwall showed that amiloride, a nonspecific NHE inhibitor, blunted the rise in intracellular sodium in ischemic isolated rat hearts [298]. However, as mentioned, amiloride is a nonspecific agent which affects other cellular processes in addition to NHE inhibition and thus, studies using this drug alone should be interpreted cautiously. However, Baartscheer and colleagues showed that cariporide markedly inhibited intracellular sodium overload and improved calcium regulation in rabbit hearts *ex vivo* in which the animals were subjected to 12 weeks of chronic combined pressure and volume overload [299].

While NHE1 activation likely represents a major mechanism for intracellular sodium elevation, other cellular processes may also contribute and should be mentioned. For example, inhibition of the late sodium current with the selective inhibitor ranolazine inhibited both hypertrophy and fibrosis as well as improving cardiac function in mice exposed to chronic pressure overload produced by aortic banding [238]. Importantly, this effect was associated with a suppression of sodium overload and improved intracellular calcium homeostasis [238].

A number of lines of evidence suggest that NHE1 is a major contributor to the development of cardiac hypertrophy and heart failure. First, cardiomyocyte hypertrophy is associated with upregulation of NHE1 expression and activity in cardiomyocytes in a number of diverse experimental models [262, 299, 300, 301, 302, 303, 304, 305] as well as in cardiomyocytes harvested from humans with end stage heart failure [306]. Secondly, many paracrine, autocrine and hormonal factors which are important initiators of the hypertrophic program are also potent activators of NHE1 activity in the heart, among these being angiotensin II, endothelin 1 and α1 adrenergic agonists [reviewed in [307]]. Indeed, paracrine and autocrine factors including angiotensin II and endothelin-1 have been shown to be key initiators of NHE1 activation (see Section 3) and the hypertrophic program following the production of myocardial stretch [308]. Thirdly, the importance of NHE1 to the development of cardiac hypertrophy and indeed myocardial remodeling and heart failure in general has been borne out by a large number of studies employing a variety of experimental models as summarized in Table 2 (Ref. [33, 220, 262, 299, 301, 302, 309, 310, 311, 312, 313, 314, 315, 316, 317, 318, 319, 320, 321, 322, 323, 324, 325, 326, 327, 328, 329, 330, 331, 332, 333]). One of the first observations documenting an antihypertrophic effect of NHE1 inhibition originated from the Karmazyn laboratory which showed that cariporide effectively attenuated early (one week) and late (twelve week) hypertrophic responses and left ventricular dysfunction following sustained coronary artery ligation in the rat [309, 310]. This laboratory further demonstrated that NHE1 inhibition also can *reverse* myocardial remodeling and heart failure when treatment is delayed for up to four weeks following coronary artery ligation [220]. The ability of NHE1 inhibition to reverse remodeling and heart failure is an important observation from a clinical standpoint. This beneficial effect has also been demonstrated in a pressure/volume overload model of heart failure in the rabbit when cariporide was started one month after the initiation of heart failure [311]. It should be noted that the salutary effects of NHE1 inhibition occurred in the absence of infarct size reduction thus demonstrating a direct antihypertrophic effect of NHE1 inhibition. It is interesting that early and transient treatment of rats with a potent NHE1-specific inhibitor for one week, followed by a five-week period in the absence of any pharmacological intervention, resulted in substantial reduction in hypertrophy and heart failure, suggesting that early activation of NHE1 following insult may be critical for the subsequent development of heart failure [312]. While NHE1 inhibition exerts antihypertrophic effects on its own, it is interesting that the benefit was enhanced with coadministration of cariporide with an angiotensin converting enzyme inhibitor (ramipril) in rats subjected to 18 weeks of sustained coronary artery occlusion [313].

**Table 2.** **Summary of studies demonstrating antihypertrophic and anti-remodeling effects of NHE1 inhibitors**.

Experimental model	NHE1 inhibitor	Reference	Main Result of inhibitor treatment
Rat 1 wk CAL	cariporide	[309]	Attenuate HY and HF
Rat 13–15 wk CAL	cariporide	[310]	Attenuate HY and HF
Rat PH/RVH	cariporide	[302]	Attenuate RV HY & fibrosis
SHR	cariporide	[33, 314, 315, 316]	Attenuate HY, apoptosis & antiarrhythmic
β1AR TG mouse	cariporide	[317]	Prevent HY, HF & fibrosis
Rabbit P/V overload	cariporide	[299, 301, 311]	↓${\mathrm{Na}}_{\text{i}}$ & ${\mathrm{Ca}}_{\text{i}}$ increases
			Attenuate HY, regress remodeling
Isoproterenol treated rats	BIIB723	[33]	Prevent HY & fibrosis
Aldosterone treated NRVM	EMD87580	[318]	Prevent HY & ${\mathrm{Na}}_{\text{i}}$ increases
Rat 12 wk CAL	EMD87580	[220]	↓& reverse remodeling & HF
Mouse 5 wk TAB	cariporide	[319]	↓remodeling, preserve systolic function
LV paced rabbits	BIIB722	[262]	↓HF & ventricle dysfunction
Rat 12 or 18 wk CAL	EMD87580	[320, 321]	Protect mito function
Hamster HHC	EMD87580	[322]	Prevent ${\mathrm{Na}}_{\text{i}}$ & ${\mathrm{Ca}}_{\text{i}}$ overload
		[323]	Prevent early death
Rat 18 wk CAL	cariporide	[313]	↑LV remodeling & ↓HY
GC-A deficient mice	cariporide	[324]	Normalize pHi, ${\mathrm{Ca}}_{\text{i}}$, HY & fibrosis
PE-treated NRVM	EMD87580	[325]	↑mito integrity & ↓ROS
ET1-treated NRVM	cariporide	[33, 326]	Prevent ET-1 HY & ↑${\mathrm{Na}}_{\text{i}}$ & ↑${\mathrm{Ca}}_{\text{i}}$
NHE1 overexpressing TG mice	cariporide	[327]	block ↑NHE1-induced ↑HY & ↑${\mathrm{Ca}}_{\text{i}}$
Estrogen treated ARVM	AVE4890	[328]	Block ↑NHE1, ↑HY & ↑${\mathrm{pH}}_{\text{i}}$
ET1-treated NRVM	AVE4890	[329]	↓ET-1-induced mito dysfunction
Glycoside-treated NRVM	AVE4890 or EMD87580	[330]	↓ induced HY
ISO-infused rats	BIIB723	[331]	↓HY & improve ${\mathrm{Ca}}^{2+}$ handling
Ang II-treated ACVM	cariporide	[332]	↓HY & ↓ROS
Rat 6 wk CAL	BIX	[312]	↓HY & ↓HF& ↓calcineurin
PE-treated NRVM	BIX	[312]	↓HY ↓HF
Ang II-treated H9c2 cells	EMD87580	[333]	↓HY & ↓cathepsin B

↑, increase; ↓, decrease; ACVM, adult cultured ventricular myocytes; Ang II, angiotensin II; β1AR TG mouse, beta 1 adrenergic receptor transgenic mouse; ${\mathrm{Ca}}_{\text{i}}$, intracellular calcium; CAL, coronary artery ligation; ET1, endothelin-1; GC-A, guanylyl cyclase-A; HF, heart failure; HY, hypertrophy; HHC, hereditary hypertrophic cardiomyopathy; ISO, isoproterenol; LV, left ventricle; MI, myocardial infarction; mito, mitochondria; ${\mathrm{Na}}_{\text{i}}$, intracellular sodium; NRVM, neonatal rat ventricular myocytes; PE, phenylephrine; PH/RVH, pulmonary hypertension with right ventricular hypertrophy; P/V, pressure/volume; ROS, reactive oxygen species; RV, right ventricle; SHR, spontaneously hypertensive rat; TAB, thoracic aorta banding.

### 7.3 NHE1 Inhibition in Different Heart Failure Models Not Involving Myocardial Ischemia

The ability of NHE1 inhibition to attenuate cardiac hypertrophy is not restricted to experimental models involving myocardial ischemia. For example, Cingolani’s group showed that cariporide reduced the hypertrophic response as well as myocardial fibrosis in the spontaneously hypertensive rat (SHR) which was dissociated from blood pressure reduction [314, 334]. Moreover, cariporide effectively prevented cardiac hypertrophy, fibrosis and left ventricular dysfunction in a transgenic mouse model overexpressing the β_1_ adrenergic receptor [317]. This study strongly suggests that NHE1 inhibition could be an effective treatment for the prevention of hypertrophy and heart failure due to increased sympathetic drive. Indeed, this is borne out by studies showing that cardiac hypertrophy and fibrosis caused by 30-day infusion of isoproterenol to rats, can be prevented by the NHE1 specific inhibitor BIIB723 [33]. Genetically induced ablation of the cardiac atrial natriuretic peptide (ANP) receptor similarly produces a cardiac hypertrophy phenotype with accompanying heart failure that can be significantly inhibited by cariporide in the absence of blood pressure reduction [324]. Hearts from these animals exhibited enhanced NHE1 activity thus suggesting that the ANP-guanylate cyclase system is an inhibitory regulator of cardiac NHE1 activity thereby mitigating the cardiac hypertrophic response to hypertension-related pressure overload [324]. In addition, cariporide inhibited the myocardial remodelling and heart failure in mice subjected to pressure overload produced by five-week thoracic aortic banding [319].

NHE1 likely plays an important role in the development of heredity hypertrophic cardiomyopathy. Bkaily’s group has shown that dietary administration of the NHE1-specific inhibitor EMD 87580 (rimeporide) prevented the development of hypertrophy, necrosis, intracellular sodium and calcium overload, as well as preventing early mortality in a dystrophic hamster model [322, 323]. Moreover, rimeporide administration to dogs with muscular dystrophy resulted in a reduction in left ventricular function deterioration in these animals [335]. Such promising results in animal models has led to clinical testing of rimeporide in young boys with Duchenne muscular dystrophy (DMD, N = 20). A phase 1B clinical trial revealed that four-week treatment with rimeporide is well tolerated and produces no safety concerns [336]. Coupled with encouraging, although preliminary biomarker data suggesting some clinical efficacy, further larger scale placebo-controlled studies are planned to demonstrate the effectiveness of rimeporide in reducing cardiomyopathy associated with DMD. The mechanisms underlying the beneficial effects of NHE1 inhibition in dystrophic cardiomyopathy are not known with certainty but, as already alluded to, likely involve attenuation of calcium and sodium overload. Moreover, as DMD is associated with mitochondrial dysfunction [337, 338], protection by NHE1 inhibitors may involve mitochondrial protection as observed in other models of heart failure (see Section 7.5).

### 7.4 Induction of Hypertrophy by NHE1 Activation

Direct effects of various hormonal as well as paracrine and autocrine factors, some of which have already been referred to, can produce hypertrophy either directly on cultured cardiomyocytes or *via* chronic *in vivo* infusion through NHE1-dependent mechanisms. Among these are aldosterone [318, 339], estrogen [328], cardiac glycosides [330], isoproterenol [331] and angiotensin II acting *via* the angiotensin ${\mathrm{AT}}_{1}$ receptor [340, 341], the effect of the latter possibly mediated by endogenous endothelin-1 and NHE1 activation [332]. These NHE1-dependent effects of pro-hypertrophic factors may be important in understanding their roles in cardiovascular diseases such as heart failure. For example, aldosterone has been shown in a landmark clinical study (the RALES study) to play an important role in development of heart failure, as demonstrated by a significant reduction in mortality and morbidity in heart failure patients treated with the mineralocorticoid receptor blocker spironolactone [reviewed in [342]]. The role of increased catecholamine drive and angiotensin II in cardiac pathology are well established and represent targets for established therapies for treating cardiovascular disorders. The deleterious effects of catecholamines on the development of heart failure are mostly mediated by β_1_ adrenoceptor activation [reviewed in [343]], indeed α_1_ blockers are generally contraindicated due to vasodilator effects of these agents resulting in reflex sympathetic activity [344]. Nonetheless, α_1_ adrenoceptor activation in the heart can produce deleterious effects such as increased cardiac fibrosis *via* calcineurin activation (of relevance see section 7.5) which would contribute to the severity of myocardial remodelling and heart failure [345] With respect to angiotensin II, targeting this hormone for the treatment of heart failure has been a mainstay for therapy for decades. This is generally achieved either by inhibition of angiotensin converting enzyme (ACE) or by the use of ${\mathrm{AT}}_{1}$ receptor antagonists (ARBs) although there is some evidence based on meta-analysis of clinical trials that ACE inhibition is more effective than ARBs in reducing mortality in heart failure patients [346]. We believe that benefit would also be achieved by NHE1-specific inhibitors. In fact, such protection could in theory be superior to that seen with targeting individual agonists as pro-remodelling effects of numerous autocrine, paracrine and hormonal factors would be inhibited by targeting NHE1. It must be added however that beneficial endogenous factors have also been identified such as insulin-like growth factor 1, which has been shown to improve cardiac function in hypertrophied hearts of spontaneously hypertensive rats while suppressing NHE1 activity [54].

A non-pharmacological mode of NHE1 upregulation involves genetic modification of the antiporter resulting in enhanced NHE1 activity. In this regard, Fliegel’s group showed that transgenic mice expressing an overactive form of NHE1 exhibit cardiac hypertrophy in the absence of any pro-hypertrophic insult, when compared to mice expressing the wild type NHE1 [64, 65]. Additionally, infection of neonatal rat ventricular myocytes with an adenoviral vector expressing a constitutively active NHE1 resulted in a hypertrophic response in the absence of any other pro-hypertrophic intervention [63].

### 7.5 Potential Mechanisms Underlying NHE1-Dependent Cardiac Hypertrophy

The mechanisms by which NHE1 activation induces cardiomyocyte hypertrophy are likely complex and associated with the stimulation of a number of intracellular pathways. As discussed above, NHE1 interacts with various intracellular cofactors and binding partners which in general, enhance the antiporter’s activity. With respect to the development of hypertrophy specifically, it has been reported that the ubiquitous multifunctional protein osteopontin originally identified in bone may be an important cofactor in mediating the hypertrophic influence of NHE1 activation. Thus, a close relationship between NHE1 and osteopontin expression was identified in cultured cardiomyocytes and silencing osteopontin in these cells supressed the hypertrophic effect of NHE1 overexpression [62]. Furthermore, osteopontin expression upregulation and the hypertrophic response in H9c2 cardiomyoblasts treated with angiotensin II was prevented by rimeporide [347].

**Fig. 6.**
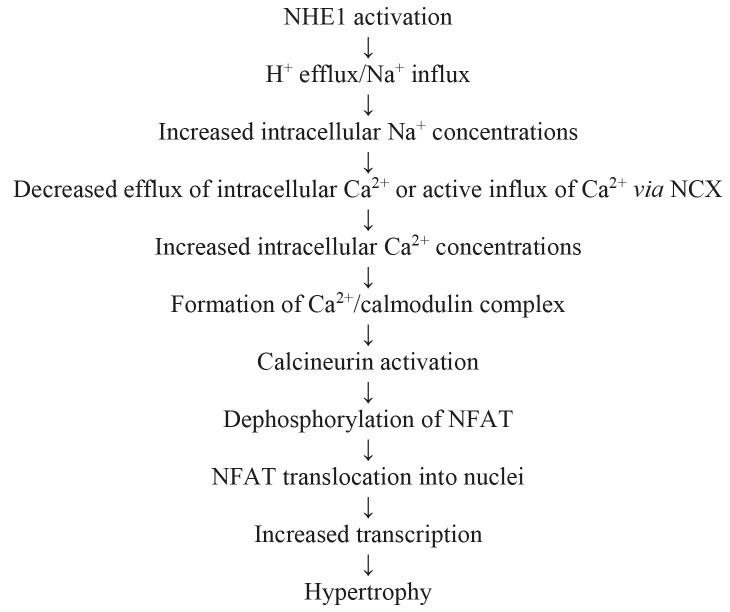
**Proposed pathway for calcineurin-mediated hypertrophy following NHE1 activation**.

Among the strongest candidates as a key factor in mediating the pro-hypertrophic effect of NHE1 activation is stimulation of calcineurin, a serine/threonine protein phosphatase which is an activator of transcriptional factors well known to be important in the pathological hypertrophic program as well as evolution to heart failure, among these being nuclear factor of activated T cells (NFAT) and myocyte enhancer factor 2 (Mef2) [348, 349]. As calcineurin can be activated by increased intracellular calcium concentrations or more specifically by formation of a calcium/calmodulin complex, it is not surprising that stimulation of NHE1 is a likely contributor to increased calcineurin activity leading to cardiomyocyte hypertrophy based on the concepts discussed in section 7.1 related to increased intracellular calcium concentrations. Indeed, overexpression of cardiac NHE1 *per se* has been shown to be sufficient to increase intracellular calcium levels, upregulate the calcineurin pathway and induce cardiomyocyte hypertrophy, thus demonstrating a strong link between NHE1 and the calcineurin pathway in promoting the hypertrophic program [327]. Inhibition of cardiac hypertrophy both *in vivo* as well as in cultured cardiomyocytes is associated with concomitant regression of calcineurin/NFAT expression [304]. Kilić *et al*. [312] showed that early and transient NHE1 inhibition was sufficient in preventing the hypertrophic response and calcineurin activation both in rats subjected to sustained coronary artery ligation as well as myocytes exposed to phenylephrine treatment. The relationship between NHE1 and calcineurin activity has also been reported with other antihypertrophic strategies including treatment with ginseng [158] or a chimeric natriuretic peptide [350]. Thus, NHE1-dependent calcineurin activation and subsequent cardiomyocyte hypertrophy likely follows the series of events summarized in Fig. 6.

The evidence for calcineurin notwithstanding, other intracellular mechanisms may also contribute to protection of the hypertrophied myocardium shown by NHE1 inhibitors particularly concerning mitochondrial preservation during the remodeling process. Recent evidence, for example, suggests that the antihypertrophic effect of NHE1 inhibition with rimeporide in cultured H9c2 cells treated with angiotensin II is associated with suppression of the activation of Cathepsin B, a cysteine protease involved in cardiovascular and other pathologies [333]. Mitochondrial protection as demonstrated by reduced mitochondrial dependent generation of reactive oxygen species may also be of importance in understanding the anti-remodeling effects of NHE1 inhibition [351]. Karmazyn’s laboratory has previously shown that rimeporide reduced MAPK activity, preserved mitochondrial membrane potential, attenuated permeability transition pore opening and reduced superoxide generation in phenylephrine-treated neonatal rat cardiomyocytes while supressing the hypertrophic response [320, 325]. Further evidence suggests that NHE1 inhibition reduces mitochondrial dysfunction in the hypertrophied heart by attenuating phosphorylation of AMP-activated protein kinase (AMPK)/glycogen synthase kinase 3β (GSK-3β) during hypertrophy both *in vivo* following sustained coronary artery ligation or in cultured myocytes in which hypertrophy was induced by endothelin-1 [329]. A potential mitochondria-related locus of protection by NHE1 inhibitors may be related to attenuation of mitochondrial fission during the post-infarction remodeling process thus preserving mitochondrial fission/fusion balance. Indeed, excessive mitochondrial fission is known to contribute to cardiac pathology associated with the development of cardiac remodeling and failure [352]. In this regard, upregulation of the primary fission protein Fis1 was significantly blunted by EMD87580 in hearts of rats subjected to 12 or 18 weeks of sustained coronary artery ligation [321]. Thus, when taken together, it appears that mitochondrial protection represents an important component underlying the salutary effects of NHE1 inhibition in cardiac hypertrophy, acting *via* multifaceted mechanisms to preserve mitochondrial integrity.

### 7.6 Anti-Remodeling Effects of Drugs Developed for the Treatment of Type 2 Diabetes: Evidence for NHE1 Inhibition 

It is interesting to note that NHE1 inhibition may also contribute to the antihypertrophic and remodelling effects of drugs not initially developed for this purpose. An example of this are the sodium-glucose cotransport 2 inhibitors, the so-called gliflozins, developed for the treatment of type 2 diabetes mellitus but which exert a number of beneficial effects on the heart not related to glucose regulation but which likely involve NHE1 inhibition [353, 354, 355, 356, 357]. For example, dapagliflozin reduced fibrosis, inflammation and left ventricular dysfunction in *db/db* diabetic mice chronically (30 days) infused with angiotensin II [358]. These beneficial effects were associated with a number of cellular effects including decreased intracellular calcium transients, decreased inflammation, decreased ROS production as well as decreased expression of the voltage-dependent L-type calcium channel and decreased NCX levels while inhibiting NHE1 [328]. The relative contribution of each of these mechanisms awaits concrete elucidation. Gliflozins, are drugs that work primarily on the kidney to aid in glucose homeostasis in diabetic patients and may have effects through NHE1 (see section 8.1). In addition to the gliflozins, teneligliptin, a dipeptidyl peptidase-4 inhibitor used for the treatment of type 2 diabetes in some countries has been shown to reduce cardiac hypertrophy and the concomitant increase in NHE1 expression in spontaneously hypertensive rats that was attributed to normalization of the elevated plasma angiotensin II levels observed in these animals [359].

### 7.7 Potential Role for Genetic Polymorphisms in Human Disease

Genetic polymorphisms of NHE1 have been reported and this includes at least one that results in human disease (recently reviewed in [22]). Briefly, the first genetic defect in humans that was found to be attributable to NHE1 was reported by Guissart *et al*. [23]. Lichtenstein-Knorr syndrome is one of several autosomal recessive cerebellar ataxias with a variety of neurological symptoms, cardiomyopathies and ataxias [360]. It is of juvenile or adolescent onset with ataxia and sensorineural hearing loss [361]. Guissart *et al*. [23] showed that the SLC9A1 (NHE1) gene, is responsible for the defect in this disease. A mutation in the SLC9A1 gene changed Gly305 to Arg. The Gly305Arg mutation causes reduced expression and decreased protein glycosylation. It also caused almost a complete absence of targeting of the protein to the cell surface and virtually no protein activity at the cell surface [23]. After this report another study [362] reported a different human mutation resulting in similar symptoms including cerebellar ataxia. In this case the mutation at amino acid Ile288 caused a premature truncation of most of the protein.

NHE1 knockout mice have also been characterized. One spontaneous mutation of NHE1 in mice was a change causing Lys442 to become a stop codon and terminate NHE1 within the transmembrane domain. Homozygous defective mice had a slow-wave epilepsy (swe) mutation. They also had an ataxic gait including locomotor ataxia that was prominent in their hind limbs Mutant homozygous mice were of small size and less than half survived to weaning [363]. A second study in mice confirmed the above physiological effects with a targeted disruption of NHE1 [364]. The genetic knockout of NHE1 allowed an interesting insight into NHE1 physiology in the myocardium. When mice with the genetic knockout of NHE1 were subjected to cardiac ischemia reperfusion injury, they were resistant in comparison to the controls [273]. This confirmed the role of NHE1 in ischemia reperfusion damage.

Various specific genetic polymorphisms have been identified in the NHE1 gene though a thorough study of their total incidence and effect on the myocardium is lacking. One polymorphism was a change of Asn266 to His [365]. The mutation was not fully characterized clinically. Mutant N226H protein was expressed and targeted properly however, the N266H protein had no detectable activity. The NHE1 cytoplasmic tail is responsible for regulation of NHE1. Another study characterized the effect of stop codon polymorphisms in the regulatory tail [366]. Stop codons at amino acid 321, 449 and 735 were examined (mutations at 321 and 449 were actually within the membrane domain). Mutants stopping at amino acids 321 and 449 lost NHE1 activity and did not target properly to the plasma membrane. They were also more rapidly degraded than wild type protein. The mutant protein ending at amino acid 735 had reduced expression and activity.

Another study [367] examined the effect of change of two polymorphisms in the phosphorylatable amino acids in the regulatory tail. It examined the Ser703 and Ser771, to proline polymorphisms [367]. Ser703 is critical to 14-3-3 binding to NHE1 and to NHE1 activation by growth factors [368, 369] (Fig. 3). Ser771 is also important in Erk 1/2 dependent activation of NHE1 [370, 371] (see section 3.1.1). The Ser703Pro mutant had virtually the same activity, targeting and expression levels as the wild type NHE1 protein. However, the Ser771Pro mutant protein had reduced activity and expression levels, but normal cell surface targeting. The Ser771Pro mutant showed abnormal regulation. It was not strongly activated by sustained intracellular acidosis, but was activated partially even by very short periods of acidosis. It was hypothesized that insertion of a Pro in this location leads to an abnormal conformation that alters synthesis or degradation of the protein and causes an abnormal regulation of the protein by conformational changes in the tail, and by the inability to be phosphorylated [367]. It is not known how mutation of these regulatory amino acids could affect NHE1 function in humans. However, Ser703 is phosphorylated by p90 ribosomal S6 kinase. A mouse heart dominant negative p90 ribosomal S6 kinase mutant was resistant to myocardial injury induced by left coronary artery occlusion [90]. One might postulate that humans carrying the Ser703Pro polymorphism might also be resistant to coronary artery occlusion, but this remains to be demonstrated.

## 8. NHE1 in Diabetes and Related Heart Diseases

Because of the ubiquitous expression of the NHE throughout the tissues and cell types of the body [1, 2] and its important function in each of these tissues, it is perhaps not surprising that changes in NHE expression and function have been implicated in a variety of diseases in a wide range of tissues and cell types. This includes brain development and function [372], dental pulp [373], immune function and inflammation [374], kidney function [375], epilepsy [363, 376], gallstone formation [377], cataracts [378] and muscular dystrophies [379]. These areas of NHE lesions have, in some cases, led to heart related problems as well. For example, it has been suggested that the heart failure associated with Becker and Duchenne muscular dystrophies may be in part due to NHE1 over-activation and the subsequent ${\mathrm{Na}}^{+}$ overload [379]. In support of this hypothesis, the chronic administration of a NHE-1 inhibitor to a dystrophic animal model prevented the intracellular ${\mathrm{Na}}^{+}$ overload and early death due to heart failure [379]. Additionally, with the predominant place inflammation and infection has in cardiovascular disease [380], it may be a particularly fruitful avenue for future research to determine if the beneficial cardiovascular effects of NHE inhibitors may be due in part to an action on the immune system and the pathways that ultimately produce inflammation. However, the two disease conditions that have been associated with NHE malfunction and may also have the most significant impact upon the cardiovascular system are diabetes mellitus and renal disease.

### Diabetes, Heart Disease and ${\mathrm{Na}}^{+}$${\mathrm{/H}}^{+}$ Exchange 

A significant change in how diabetic heart disease was viewed was first proposed with experimental evidence in the 1970’s and early 1980’s [381, 382, 383, 384, 385, 386, 387, 388, 389]. Instead of diabetic heart disease and failure being viewed as a primarily vascular lesion [390], the aforementioned works clearly identified a subcellular basis for a cardiomyopathy independent of vascular complications. The cardiac dysfunction was demonstrated in both insulin dependent and non-insulin dependent models of diabetes [382, 383, 391, 392].

Insulin dependent models of diabetes exhibited hearts resistant to ischemic/reperfusion insult [393]. A reduced ${\mathrm{[pH]}}_{\text{𝑖}}$ recovery was found in response to an acid load in cardiac muscle preparations from insulin deficient diabetic animals [394]. This suggested that a decrease in NHE activity or expression levels was present in the diabetic heart. A direct demonstration of a reduced ${\mathrm{Na}}^{+}$${\mathrm{/H}}^{+}$ exchange activity was found in cardiac sarcolemmal vesicles isolated from diabetic animals in comparison to control preparations [395]. However, expression levels of NHE1 mRNA were unchanged in hearts from streptozotocin-induced diabetic rats [396]. The decrease in ${\mathrm{Na}}^{+}$${\mathrm{-H}}^{+}$ exchange in the heart would be expected to lessen the influx of ${\mathrm{Ca}}^{2+}$_i_, much as a drug that inhibits NHE, as discussed earlier in this manuscript. This, in turn, would result in a protection of the diabetic heart from ischemic/reperfusion injury.

However, whereas type 1 diabetic animals are more resistant to ischemia, insulin resistant Type 2 diabetic animals were conversely more sensitive to ischemic challenge. The decreased post-ischemic cardiac performance exhibited by hearts from late-stage insulin-resistant models of diabetes, may be due to greater endogenous stores of glycolytic substrates and the resultant excessive production of lactate and $H^{+}$ [397]. This would tend to enhance the exchange of ions through the NHE pathway and generate augmented cardiac damage [398]. This enhanced NHE activation in Type 2 diabetes agrees well with its activation during hyperinsulinemia [399]. Packer [400, 401] has proposed a key role of cardiac and vascular NHE1 as well as renal NHE3 as principal factors linking diabetes with the development of heart failure. Thus, it was proposed that neurohormonal-dependent upregulation of NHE1 and NHE3 would result in NHE1-dependent cardiac remodelling coupled with NHE3-dependent renal sodium retention, the combination accelerating the progression to heart failure [401].

As noted above in section 7.6, sodium/glucose cotransporter 2 (SGLT2) inhibitors, gliflozins, are drugs that work primarily on the kidney to aid in glucose homeostasis in diabetic patients. Empagliflozin has been reported to attenuate both sodium and calcium dysregulation in mouse ventricular myocytes treated with ouabain potentially *via* NHE1 inhibition [402]. It is also important to note that empagliflozin has recently been shown to reduce oxidative stress in cultured human umbilical vein endothelial cells and coronary artery endothelial cells through a mechanism involving NHE1 inhibition [403] thus providing further supporting evidence for the beneficial effects of gliflozins on the heart through NHE1 inhibition. However, several recent studies have suggested that these compounds also inhibit cardiac NHE1 activity [353, 354, 358, 404] and expression [358]. This inhibition appears to be the mechanism for a lowering of cytosolic ${\mathrm{Na}}^{+}$, vasodilation [354], a decrease in lactate generation [404], an attenuation of the diabetic cardiomyopathy [358] and a reduction in infarct size in the post-ischemic reperfused heart [405]. This inhibition of NHE1 by SGLT2 inhibitors appears to be a class action effect as empagliflozin, dapagliflozin and canagliflozin have been reported to inhibit NHE [358]. It should be noted however that there is controversy in this area and there are reports that SGLT2 blockers do not directly inhibit NHE1 [406, 407]. The different results reported may be due to species differences or due to the method of drug application. Indeed, in a recent report [408] the long-term treatment of H9c2 cells with empagliflozin was shown to inhibit expression of the NHE1 protein while short term treatment did not inhibit NHE1 expression. This also raises the possibility that some of the effects observed are due to inhibition of protein expression, rather than a direct inhibitor effect on the protein.

The glifozins are not the only non-specific drug interactions that may have their biological action *via* a primary effect on the NHE. NHE3 in the kidney is affected by incretin-based agents, antagonists of the renin-angiotensin system, insulin and insulin sensitizers, statins and spironolactone [409].

## 9. The Effects of NHE1 Inhibitors in Clinical Settings

The robust experimental data demonstrating substantive cardioprotective properties of NHE1 inhibitors, unmatched by other cardioprotective strategies, rapidly progressed to clinical evaluation of NHE1 inhibitors in patients with coronary artery disease, mostly employing cariporide as the drug of choice. The first such study recruited a total of 100 patients who had experienced an acute myocardial infarction (AMI) and who were subjected either to percutaneous transluminal coronary angioplasty (PTCA) with cariporide administered at the time of reperfusion or with a placebo [410]. Patients receiving cariporide exhibited improved left ventricular function three weeks post-PTCA and reduced plasma enzyme levels within 72 hours after reperfusion, the latter indicating an inhibition of reperfusion injury by cariporide [410].

The results of the above study were somewhat surprising in view of the small number of patients recruited but also because experimental studies have demonstrated that optimal protection by NHE1 inhibitors occurs when the drug is also present during the ischemic period before the onset of reperfusion. Clinically, this can be achieved under controlled I/R conditions such as in coronary artery bypass grafting (CABG, discussed below). Indeed, a Phase II clinical study named “Evaluation of the Safety and Cardioprotective Effects of Eniporide in AMI” (ESCAMI) study was conducted to investigate the hypothesis that eniporide would reduce injury given as an adjunct to reperfusion performed either by thrombolysis or PTCA [411]. This was a phase 2 international multicenter randomized, double-blinded, placebo-controlled, dose-finding trial which was carried out in two stages: in Stage 1 (433 patients), eniporide was administered at 50 mg, 100 mg, 150 mg or 200 mg whereas in Stage 2 (978 patients), based on the results of Stage 1 eniporide was further studied at doses of 100 mg or 150 mg. For both stages, eniporide or placebo was administered over a 10-minute infusion period. Specifically, in patients subjected to thrombolytic therapy, infusion was completed at least 15 minutes after starting thrombolytic treatment, whereas in angioplasty, the patient’s infusion was completed at least 10 minutes before the start of PTCA. The results of the Stage 1 study were encouraging in that there were significant reductions in infarct size, the primary efficacy end point of the study, of 25.7% and 41.7%, were found with 100 mg and 150 mg eniporide, respectively. This effect was more evident in the PTCA treated patients. However, no protection was observed in Stage 2 of this trial using these two eniporide doses. It is interesting to add that in a subgroup of over 300 patients who were subjected to delayed reperfusion (>4 hours after onset symptoms) a significant reduction in heart failure symptoms was observed in the 150 mg eniporide group when compared with placebo (placebo 21.9%, eniporide 11.1%).

Further evaluation of the protective effects of NHE1 inhibitors was then carried out in two important clinical trials. The first of these was the Guard During Ischemia Against Necrosis (GUARDIAN) trial which was designed to determine whether cariporide could reduce mortality and MI in patients at risk of myocardial necrosis as well as to determine the drug’s safety [412]. GUARDIAN was a combined phase 2/phase 3 international multicenter, double-blind, randomized and dose-finding study in which the primary objective was to evaluate the efficacy of cariporide in reducing all-cause mortality and/or MI across the various entry populations 36 days after randomization. This study recruited 11,590 patients who were either hospitalized for an acute coronary syndrome (unstable angina or non–Q-wave myocardial infarction) or who were subjected to either PTCA or CABG. Patients were randomized to receive either one of three cariporide doses of 20 mg, 80 mg or 120 mg or placebo which were administered every eight hours for two to seven days as a 60-minute infusion. Starting time for cariporide administration differed based on the underlying condition and as decided by individual investigators. Generally, cariporide was initiated as soon as possible after admission in patients with acute coronary syndrome and between 15 minutes and 2 hours before PTCA or CABG. Doses of 20 and 80 mg were ineffective across all clinical settings. However, at day 36 CABG patients who were treated with 120 mg cariporide exhibited a significant 25% risk reduction in either death or myocardial infarction which primarily reflected a 32% risk reduction in nonfatal infarctions.

The GUARDIAN study, while showing no overall benefit of cariporide when assessed across all clinical settings, did demonstrate substantial benefit, as noted above, with fewer end-point events when administered at the 120 mg dose to high-risk CABG patients. This encouraging result was subsequently used as the major basis for the phase 3 ${\mathrm{Na}}^{+}$${\mathrm{/H}}^{+}$ Exchange inhibition to Prevent coronary Events in acute cardiac condition (EXPEDITION) trial to study the potential benefit of cariporide on death and non-fatal myocardial infarction in CABG patients [413]. Thus, in this trial a total of 5761 patients were randomized to receive intravenous cariporide as a 180 mg 1-hour preoperative loading dose followed by 40 mg per hour over a 24 hour and then by 20 mg per hour over the subsequent 24 hours, or placebo. The primary composite endpoint of death or MI in the EXPEDITION study was assessed at 5 days with patients followed for up to 6 months.

The results from the EXPEDITION trial were promising *vis a vis* the cardioprotective effects of cariporide in that the incidence of death or MI was reduced from 20.3% in the placebo group to 16.6% in patients treated with cariporide as was the incidence of MI alone (18.9% in the placebo group vs 14.4% in cariporide-treated patients), both highly significant reductions. These beneficial effects were maintained at 6 months follow up. Unfortunately, the beneficial cardiac effects of cariporide were associated with increased mortality from 1.5% in the placebo group to 2.2% with cariporide. These increases were statistically significant at 5 days and 3 months follow up but not at 6 months and were caused almost exclusively by a significantly higher incidence of thromboembolic strokes in patients receiving cariporide [413].

## 10. Perspectives and Future Directions 

This paper has presented an overview of NHE1 in terms of its chemistry, regulation and its role in cardiac pathologies, the latter pertaining primarily to myocardial ischemic and reperfusion injury as well as myocardial remodeling resulting in heart failure. Much has been learned about the role of the antiporter as a critical regulator of intracellular pH but of greater relevance to the present discussion, its potential as a target for pharmacological intervention for cardiac therapeutics. The robust experimental evidence demonstrating salutary effects of NHE1 specific inhibitors has led to a rapid evaluation of these agents in the clinical setting particularly with respect to the assessment of cariporide as a cardioprotective agent in patients subjected to reperfusion protocols. Needless to say, the overall results seen in clinical trials with cariporide, as well as with eniporide, have been disappointing as evidenced by lack of efficacy and unexpected side effects as seen in the EXPEDITION study (even though a cardioprotective influence was demonstrated). A thorough evaluation of these results has previously been presented [414] and will, therefore, be discussed briefly here.

The results of the initial small study [410] notwithstanding, the failure to demonstrate efficacy in either the ESCAMI study or in either the thrombolysis or PTCA arms in the GUARDIAN trial, is surprising as animal data clearly demonstrated optimal protective efficacy of NHE1 inhibitors when the drug is present during the ischemic period, as noted in Sections 6.2 and 6.3. Indeed, when CABG patients were treated prior to surgery with the highest cariporide dose (120 mg group) in the GUARDIAN study, significant cardioprotection was observed [412]. Thus, expectations were high for favorable results in EXPEDITION as only CABG patients were recruited to this study with one standard cariporide dosing regimen. In fact, a significant cardioprotection was seen in EXPEDITION but the results were associated with a significantly increased incidence of ischemic strokes, thus resulting in an early cessation of the trial. The reasons for the increased incidence of strokes in cariporide-treated patients are not known with certainty. Importantly, we do not know for example whether this reflects a property of NHE1 inhibitors in general or cariporide specifically. The former is unlikely as NHE1 inhibitors have been extensively shown to exert cerebral protective effects and have been proposed as a potential treatment for strokes [415]. In addition, inhibitors of platelet NHE1 inhibit platelet aggregation [416].

There is a strong possibility that the increased incidence of strokes seen in EXPEDITION reflected the substantially different dosing regimen as well as total dose of cariporide administered when compared to that administered in the 120 mg CABG group in the GUARDIAN study. Thus, the total cariporide administered to patients in EXPEDITION was 1620 mg over a 48-hour period compared to 720 mg during the same time period in the GUARDIAN study. It is therefore possible, or even likely, that the increased incidence of strokes seen in cariporide-treated patients in EXPEDITION reflected an unnecessary overdosing with cariporide, particularly when compared with the highest dose CABG group in the GUARDIAN study which resulted in cardioprotection but no increase in the incidence of strokes [412]. As the treatment regimen in the highest cariporide dose CABG group in GUARDIAN resulted in cardioprotection, it is difficult to rationalize the more than two-fold increase in cariporide dosing in EXPEDITION during the pre-surgery 48-hour period. As outlined by the late Dr Gerald Buckberg, a renowned cardiac surgeon who was a participating investigator and a member of the EXPEDITION Steering Committee, the sponsoring company of the trial altered the steering committee’s recommendations regarding dosing by extending drug delivery duration and adding a high dose delivery. “They did this despite our emphasizing that these patients did not need elevated doses” [417].

The increased incidence of cerebrovascular events seen in EXPEDITION had a profoundly negative effect on the clinical development of NHE1 inhibitors as evidenced by a total cessation of all NHE1 inhibitor-related research and development by the pharmaceutical industry. Whether this action was justified is difficult to address as there is a complete paucity of data in the literature addressing the question of a possible pro-thrombotic effect of cariporide specifically or NHE1-specific inhibitors in general. For example, was the thrombotic effect of cariporide due to high dosing and can this be confirmed in the laboratory setting? Are there any insights into potential mechanisms for the pro-thrombotic effect of cariporide and does this involve NHE1 inhibition or non-specific effects of cariporide? This question would be particularly important to address since NHE1 activity likely contributes to platelet aggregation with an attenuation of the latter by NHE1 inhibitors, as already noted above. Do other NHE1-specific inhibitors share this pro-thrombotic effect particularly at high doses? Does the method of administration influence the deleterious effect of cariporide? In this regard, cariporide and other NHE1 inhibitors would obviously be most effective as cardioprotective agents in CABG patients, based on existing clinical data with cariporide. Would an oral preparation be effective in producing cardioprotection and would this minimize any possible prothrombotic risk compared to drug infusion? In view of the immense potential of NHE1 inhibitors for the treatment of heart disease as outlined in this review, the results of the EXPEDITION study should likely not have precluded the development of newer NHE1-specific inhibitors without addressing the issues just raised. The potential for NHE1 inhibitors to benefit patients with cardiovascular disorders warrants further research and possible clinical development of these agents. As stated by the EXPEDITION study authors “the use of NHE inhibitors could lead to significant improvement in medium- and long-term survival among patients undergoing heart surgery as well as those at risk of MI at any time” [413]. This potential benefit should not go unexplored.

With the recent elucidation of the 3D structure of NHE1 [11] and the development of novel inhibitors towards NHE1, there are also new opportunities. Understanding the precise location of the inhibitor binding pocket and its coordination may allow medicinal chemists to design novel inhibitors with even higher specificity and potency towards NHE1. Additionally, some such novel NHE1 inhibitors have recently been developed [226, 227, 228, 230] though they have not been tested in a cardiovascular disease setting. The advantage of novel inhibitors that are structurally different from cariporide is that they may avoid the stigma of the previous problems and it may be easier to obtain regulatory approval for them. There has also been little attention to altering NHE1 regulation in the disease state, which is another approach that has shown some promise [66, 83, 90], but has not been further developed in the clinic.

## 11. Conclusions

In summary, we propose that NHE-specific inhibition remains a worthwhile endeavour in the development of effective therapeutics for the treatment of heart disease. The scientific evidence for an effective approach to mitigating damage to the myocardium remains very strong as is the concept of NHE1 inhibition for the treatment of heart failure. Despite concerns with earlier clinical trials, the rationale and approach remain sound and new compounds for proposed treatment along with a more careful application based on experimental data could lead to useful clinical treatments for this major health problem.
